# *Pseudomonas donghuensis* HYS virulence towards *Caenorhabditis elegans* is regulated by the Cbr/Crc system

**DOI:** 10.1038/s41598-019-45145-8

**Published:** 2019-06-19

**Authors:** Guanfang Xie, Man Zeng, Jia You, Zhixiong Xie

**Affiliations:** Hubei Key Laboratory of Cell Homeostasis, College of Life Sciences, Key Laboratory of Analytical Chemistry for Biology and Medicine (Ministry of Education), Wuhan University, Wuhan, 430072 P.R. China

**Keywords:** Bacterial pathogenesis, Bacterial genetics

## Abstract

*Pseudomonas donghuensis* HYS is the type strain of a recently identified species, *P*. *donghuensis*, which has pathogenic potential with an unclear virulence mechanism. In this study, we used *Caenorhabditis elegans* as a host to explore the virulence mechanism of *P*. *donghuensis* HYS. Based on a correlation between *P*. *donghuensis* HYS virulence and its repellence property, we identified 68 potential virulence-related genes, among them the Cbr/Crc system, which regulates the virulence of prokaryotic microorganisms. Slow-killing assays indicated that *cbrA*, *cbrB*, or specific sRNA-encoding genes all affected *P*. *donghuensis* virulence positively, whereas *crc* affected it negatively. Transcriptome analyses demonstrated that the Cbr/Crc system played an important role in the pathogenesis of *P*. *donghuensis*. In addition, experiments using the worm mutant KU25 *pmk-1*(km25) showed a correlation between *P*. *donghuensis* HYS virulence and the PMK-1/p38 MAPK pathway in *C*. *elegans*. In conclusion, our data show that Crc plays a novel role in the Cbr/Crc system, and the *P*. *donghuensis* virulence phenotype therefore differs from that of *P*. *aeruginosa*. This process also involves *C*. *elegans* innate immunity. These findings significantly increase the available information about Cbr/Crc-based virulence mechanisms in the genus *Pseudomonas*.

## Introduction

Virulence is a special survival strategy for pathogens and involves nutrient competition and self-protection under harsh conditions protecting them from hostile circumstances such as predation and helping them resist host defences when encountering unavoidable attacks^1,2^. When dealing with hosts, pathogens must respond rapidly to adverse situations, activate associated virulence-related programmes, and manage host immune attacks^3^. All these processes must be executed by expressing a series of genes, which requires a sophisticated regulatory system^4^.

The genus *Pseudomonas* is ubiquitously distributed and includes many pathogens, such as *P*. *fluorescens*, *P*. *syringae*, *P*. *putida*, and *P*. *aeruginosa*^5–8^. The pathogenicity of *P*. *aeruginosa* has been researched to the greatest extent. It has a large arsenal of virulence determinants and can serve as a reference for other studies of virulence mechanisms in *Pseudomonas*. *P*. *aeruginosa* possesses many regulatory systems, and the major systems for environmental adaptation are two-component systems, including the GacS network, the Roc network, the Rcs/Pvr network, the PhoQP- and PmrBA-involving network, the Chp pathway, the FimS/AlgR network, the Wsp pathway, and the CbrA/CbrB system^9–11^.

*Pseudomonas donghuensis* is a recently identified species of this genus^12^. This species antagonizes bacteria, fungi, and oomycetes, suggesting its potential as a pathogen^13,14^. However, the pathogenic mechanism of *P*. *donghuensis* remains poorly understood. *P*. *donghuensis* HYS is the type strain of this new species, and it can therefore be used to represent the whole species when conducting pathogenic research. In addition, this strain has its own characteristics, such as production of a large amount of siderophores, including pyoverdine^15^. In our previous study, this strain was more virulent towards *Caenorhabditis elegans* than *P*. *aeruginosa* PA14, suggesting the possible existence of a new pathogenic mechanism. For a potential pathogen with such unusual characteristics, the known pathogenic mechanisms are of little use as a reference, and the most effective method to determine its mechanisms is to use a suitable model and conduct direct screening by constructing a mutant library.

*C*. *elegans* is suitable for high-throughput screening. It not only provides a whole-body system but also produces a large number of progeny. In addition, this worm can interact with many known human pathogens^16^. In our previous experiment, *P*. *donghuensis* HYS caused a high level of *C*. *elegans* death in a slow-killing experiment, and during this process, a relationship was observed between *P*. *donghuensis* HYS virulence and its repellence of *C*. *elegans*.

In this study, we tested the correlation between *P*. *donghuensis* HYS virulence and its repellence of *C*. *elegans*, which we used to conduct a choice assay to screen potential virulence-related genes. In addition, we further investigated these genes through survival evaluation. Our work explored the function of the Cbr/Crc system in virulence regulation in *P*. *donghuensis* HYS. We found that except for Crc, which had a negative effect on virulence, all other components of the system increased virulence. Transcriptomic analyses showed the crucial role of the Cbr/Crc system among the remaining screened genes. In addition, we tested the correlation between *P*. *donghuensis* HYS virulence and *C*. *elegans* innate immunity. Our data demonstrate that the Cbr/Crc regulatory system plays a crucial role in virulence regulation in *P*. *donghuensis* HYS. These results may facilitate comprehension of the pathogenesis of *P*. *donghuensis* as well as the genus *Pseudomonas*.

## Results

### *P*. *donghuensis* HYS repels *C*. *elegans* and causes worm death in a slow-killing assay

**Figure 1:**
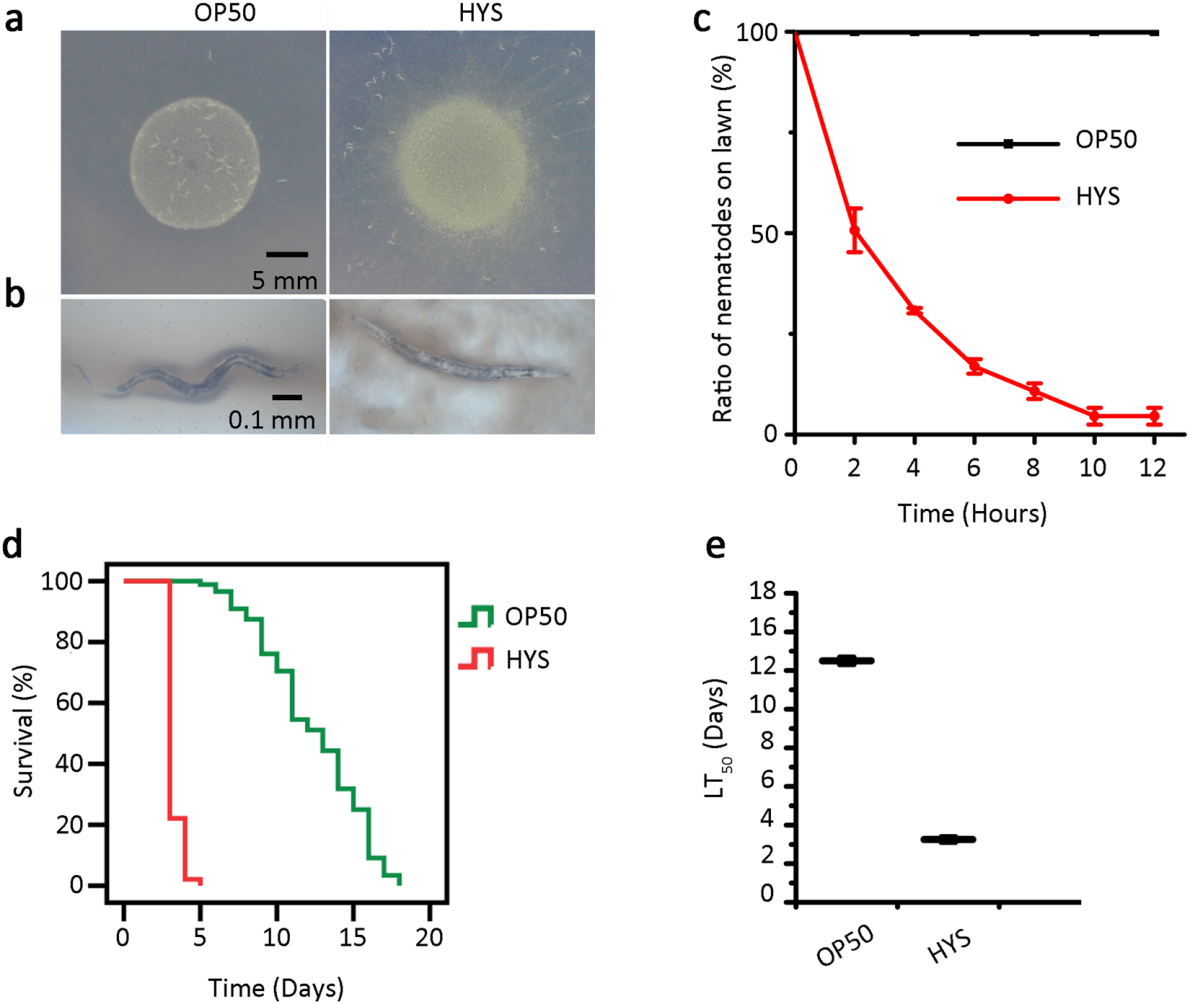
Correlation between the repellence and virulence of *P*. *donghuensis* HYS towards *C*. *elegans*. (**a**) Distribution and (**b**) final state of the worms exposed to a bacterial lawn of *E*. *coli* OP50 or *P*. *donghuensis* HYS after 12 hours of incubation. (**c**) The distribution of worms on bacterial lawns recorded every 2 hours for a total of 12 hours. n = 3. Proportion of worms on the lawn = number of worms on the tested bacterial lawn/number of worms on the entire agar plate. (**d**) Survival curves of worms fed *E*. *coli* OP50 or *P*. *donghuensis* HYS. The curves are representative of three independent experiments. (**e**) The average LT_50_ value was used to evaluate the living condition of worms on the tested strain.

*P*. *donghuensis* HYS had strong repellence and virulence towards *C*. *elegans*. In the food-avoidance experiment shown in Fig. 1a, worms remained inside the bacterial lawn of *E*. *coli* OP50, but they were outside the bacterial lawn of *P*. *donghuensis* HYS after 12 hours. This repellence was consistent throughout the observation period (Fig. 1c. n = 3). We also observed that worms that failed to leave the *P*. *donghuensis* HYS lawn within this time moved more slowly or were dead (Fig. 1b). A slow-killing assay was performed to test bacterial virulence against *C*. *elegans*. As shown in Fig. 1d,e, worms exposed to *E*. *coli* OP50 had a normal life span of nearly three weeks, and the average LT_50_ value (the time required to kill 50% of the nematodes) was 12.51 ± 0.20 days (n = 3). By contrast, *P*. *donghuensis* HYS killed worms in 5 days, and the average LT_50_ value was 3.25 ± 0.15 days (n = 3). This virulence is stronger than that of PA14 or PAO1 previously reported^17,18^. Repellence of worms by *P*. *donghuensis* HYS was also observed during the killing process. These results show that we could investigate the virulence of *P*. *donghuensis* HYS through nematode preference.

### Identification of potential virulence-related genes in *P*. *donghuensis* HYS

The feeding preference of *C*. *elegans* can be used to identify potential virulence genes in *P*. *aeruginosa* PAO1^19^. Therefore, after observing the correlation between *P*. *donghuensis* HYS repellence and its virulence, we performed a choice assay to evaluate the repellence of *P*. *donghuensis* HYS towards *C*. *elegans*. The results showed that worms were strongly repelled by *P*. *donghuensis* HYS (choice index = −0.91 ± 0.04, n = 10) when choosing between lawns of *E*. *coli* OP50 and *P*. *donghuensis* HYS. Since such bacterial repellence reflects the preference of *C*. *elegans* for bacteria, we screened for potential virulence-reduced mutants by measuring their repellence ability.

**Figure 2:**
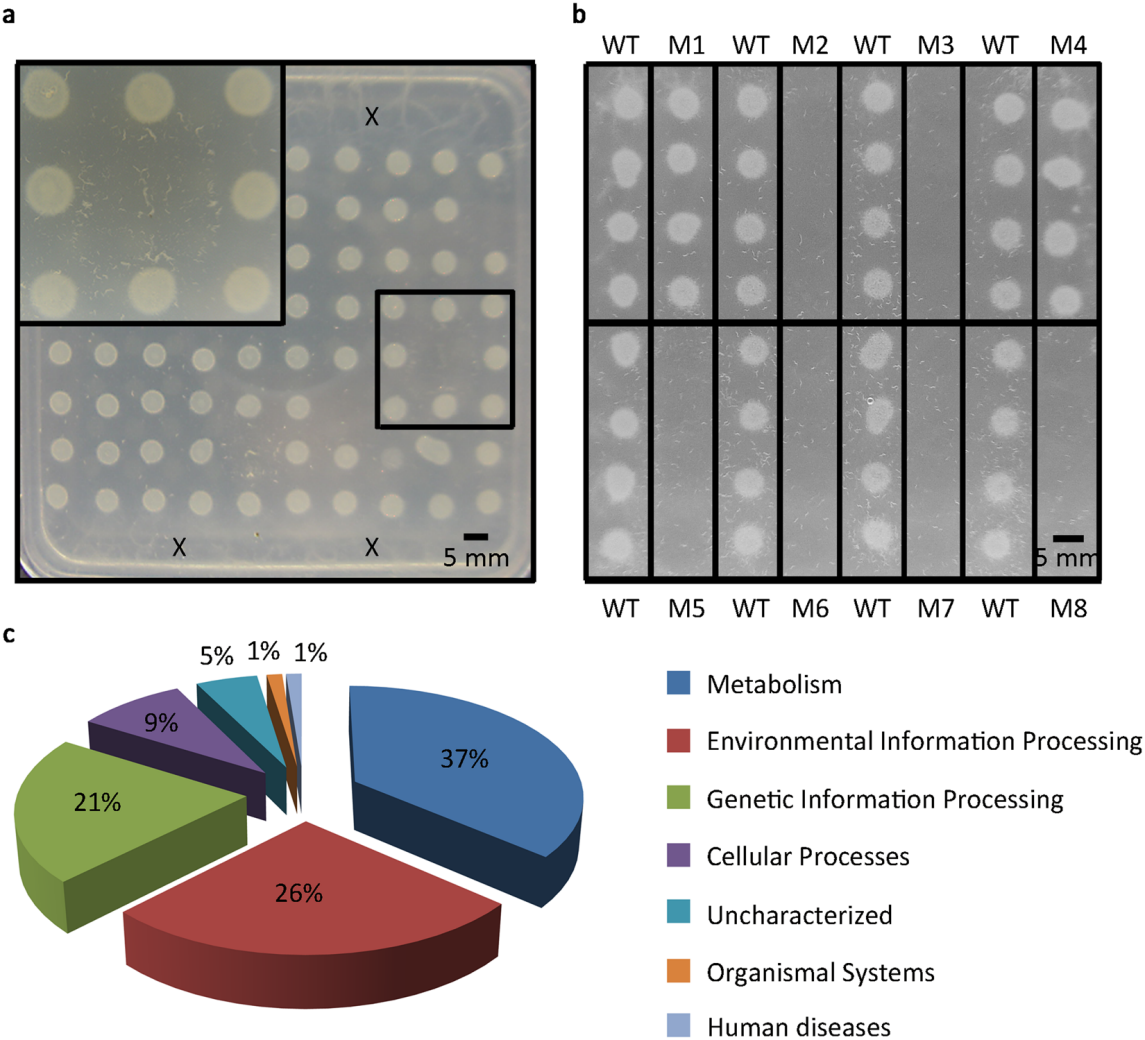
Screen for *P*. *donghuensis* HYS mutants demonstrating reduced repellence of *C*. *elegans* and pathway classifications of the identified genes. (**a**) Primary screen for mutants with reduced repellence. Each round lawn represents one bacterial insertion mutant, and the selected mutants are shown at 2× magnification in the upper left corner. (**b**) Secondary screen of mutants with reduced repellence that were selected in the primary screen. Lawns in a row inside the same rectangular box represent four different clones of the same mutant. X shows the position of the *E*. *coli* OP50 lawn, and worms were placed around it. The missing lawn represents repellence-reduced mutants. Scale bars, 5 mm. (**c**) Pathway classifications of the identified genes.

In total, 17,843 transposon insertion mutants were constructed using the vector pBT20 and then tested. This number is more than three times the number of putative genes in the *P*. *donghuensis* HYS genome. The bacterial repellence of worms was utilized for large-scale primary screening. As shown in Fig. 2a, absent lawns were consumed by *C*. *elegans*, and these mutants were considered to show reduced repellence for secondary screening. As shown in Fig. 2b, lawns in the same frame represent four different colonies of the same mutant. The mutants absent from the agar plates (M2, M3, M5, M6, M7, and M8) exhibited stably reduced repellence characteristics and were reserved for further investigation, while those remaining on the plates (M1 and M4) were still repellent to worms and were not studied further. After screening, we used thermal asymmetric interlaced (TAIL)-PCR and arbitrarily primed PCR to obtain the transposon-flanking sequences of the mutants, with the transposon insertion sites determined by comparison with the draft genome sequence of *P*. *donghuensis* HYS.

After identification, 68 *P*. *donghuensis* HYS transposon insertion sites were obtained, accounting for 0.38% of the total insertion mutant sites. The corresponding affected genes were considered to be relevant to bacterial repellence and were also investigated for bacterial virulence. As shown in Supplementary Table S1, the identified genes were scattered throughout the *P*. *donghuensis* HYS genome and were not noticeably associated with gene clusters. Therefore, we attempted to identify the pathways to which these genes contributed. Of the 68 identified genes, 29 participated in metabolism, 21 were related to environmental information processing, 17 were related to genetic information processing, 7 were involved in cellular processes, 1 was involved in organismal systems, 1 was relevant to human disease, and 4 encoded proteins that had not yet been characterized (Fig. 2c). The pathway classifications are shown in Supplementary Table S2.

Among the 68 identified gene sites, only two sequences (*UW3_RS0113375* and *UW3_RS0113380*) were located adjacent to each other. These genes were related to regulatory systems and were categorized under environmental information processing. To identify these two genes, we searched for and analysed their products (the results are shown in Supplementary Table S3). The product of *UW3_RS0113375* was identified as CbrB, and that of *UW3_RS0113380* was identified as CbrA. These two proteins are parts of the same two-component system, and CbrA is reportedly involved in virulence and virulence-related processes via CbrB^11^. Insertion mutants of these two genes were selected first by *C*. *elegans*, as they were completely consumed by *C*. *elegans* in the shortest length of time, indicating the crucial role of this two-component system in the virulence of *P*. *donghuensis* HYS. In addition, the global regulator Crc, which is involved in the same system, functions in bacterial virulence^20^. Therefore, we selected relevant genes from the CbrA/CbrB system for further investigation.

### The virulence of *P*. *donghuensis* HYS is regulated by the CbrA/CbrB/CrcZ/CrcY system in a *C*. *elegans* slow-killing assay

**Figure 3:**
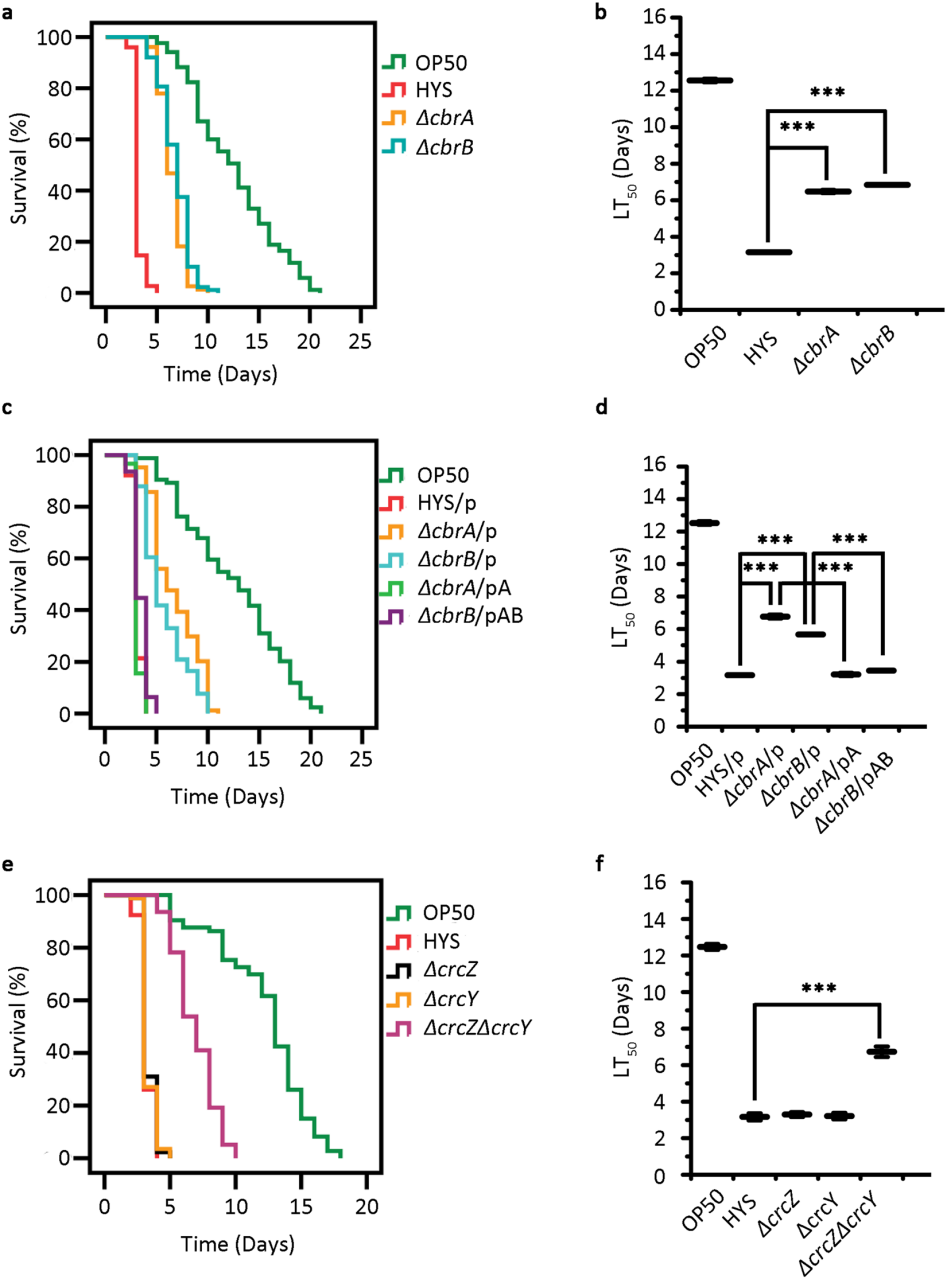
The CbrA/CbrB/CrcZ/CrcY system regulates virulence in *P*. *donghuensis* HYS. (**a**,**b**) The functions of *cbrA* and *cbrB* in *P*. *donghuensis* HYS virulence were assessed based on the survival curves and LT_50_ values of gene deletion mutants. (**c**,**d**) Their functions were further confirmed by using gene complementation strains. p represents the expression plasmid pBBR1MCS-2, pA represents the recombinant plasmid pBBR2-*cbrA*, and pAB represents the recombinant plasmid pBBR2-*cbrAB*. (**e**,**f**) Small RNAs were also tested in the slow-killing assay. Curves are representative of three independent experiments. Data are presented as the mean ± standard deviation from three independent experiments. ***p < 0.001 Student’s t-test.

In the previous screen, transposon insertion mutations in both the *cbrA* and *cbrB* genes impaired the repellence of *P*. *donghuensis* HYS. To test the functions of these two genes in virulence, we constructed *cbrA* and *cbrB* deletion mutants (*ΔcbrA* and *ΔcbrB*, respectively) and performed a slow-killing assay utilizing *C*. *elegans*. Survival analysis showed that both the life span and the LT_50_ value of *C*. *elegans* fed deletion mutants were approximately twice as long as those fed the wild-type strain (LT_50_ values increased from 3.15 ± 0.01 days to 6.48 ± 0.09 and 6.85 ± 0.02 days, respectively. n = 3) (Fig. 3a,b). In addition, we tested the growth of the mutants, which exhibited growth curves similar to that of the wild-type strain (Supplementary Fig. S1. n = 3). We also tested growth of complemented strains of both *ΔcbrA* and *ΔcbrB* strains, and found similar growth tendency (Supplementary Fig. S2. n = 3). Thus, the reduced virulence was not due to a deficiency in bacterial growth, since large amounts of bacteria were observed after the slow-killing assay was completed, showing that the *ΔcbrA* and *ΔcbrB* strains had reduced virulence. Restoration of virulence in the complemented strains further confirmed the functions of these two genes in bacterial virulence, as shown in Fig. 3c,d (LT_50_ values decreased from 6.76 ± 0.12 and 5.66 ± 0.02 days to 3.23 ± 0.07 and 3.13 ± 0.08 days, respectively. n = 3).

To determine which small RNAs were present in the genome of *P*. *donghuensis* HYS, we conducted a search. As shown in Supplementary Fig. S3, two small RNAs were detected and identified as CrcZ and CrcY. The gene encoding CrcZ was located between the genes *cbrB* and *pcnB*, as described in PAO1^21^. This gene showed 88% identity with *P*. *aeruginosa crcZ* and contained six AANAANAA boxes for Crc binding. The gene encoding CrcY was located between the genes *UW3_RS0124555* and *UW3_RS0124560*, and it also contained six AANAANAA boxes. To identify the function of CrcZ and CrcY in virulence, we constructed single-knockout mutants of each gene and a double-knockout mutant of both genes. Only the double-knockout mutant exhibited reduced virulence to *C*. *elegans*, and the LT_50_ value increased from 3.17 ± 0.20 days to 6.73 ± 0.29 days. n = 3. (Fig. 3e,f), revealing the redundant roles of these two small RNAs. These results show that the virulence of *P*. *donghuensis* HYS was regulated by the CbrA/CbrB/CrcZ/CrcY system.

### The *crc* gene plays a negative role in regulating the virulence of *P*. *donghuensis* HYS

**Figure 4:**
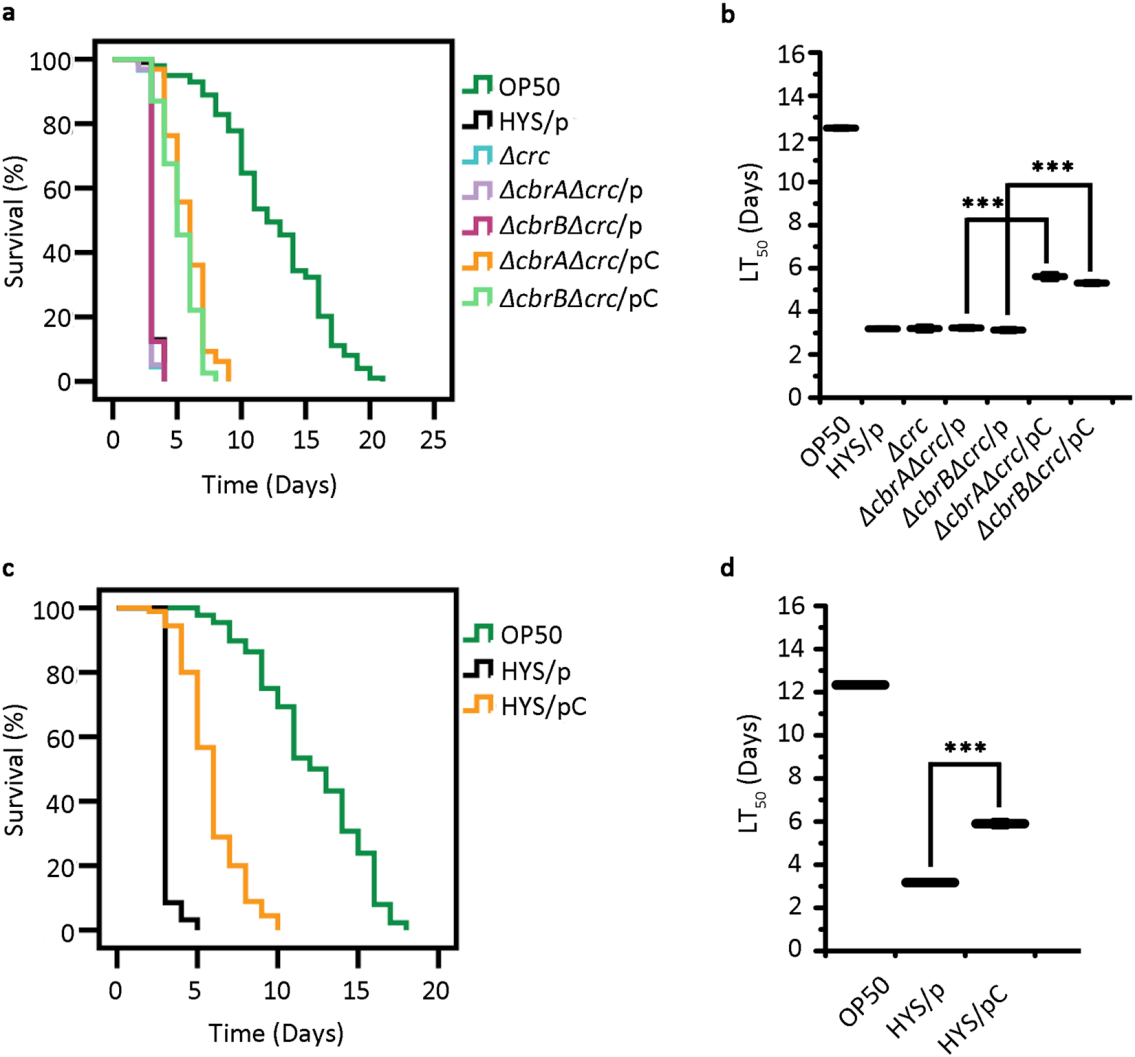
The negative effects of *crc* on *P*. *donghuensis* HYS virulence. (**a**,**b**) The effects of *crc* were assessed based on survival curves and LT_50_ values of the double-knockout mutants *ΔcbrAΔcrc* and *ΔcbrBΔcrc*, and its function was further confirmed using *crc* gene complementation strains. (**c**,**d**) Overexpression of *crc* in the wild-type strain was also investigated to assess virulence. p represents the expression plasmid pBBR1MCS-2, and pC represents the recombinant plasmid pBBR2-*crc*. Curves are representative of three independent experiments. Data are presented as the mean ± standard deviation from three independent experiments. ***p < 0.001 Student’s t-test.

To study the function of the gene *crc*, we first constructed a *crc* deletion mutant and conducted a slow-killing assay in which no reduction in virulence was observed (the LT_50_ value of *P*. *donghuensis* HYS/p was 3.20 ± 0.06 days, and the LT_50_ value of *Δcrc* was 3.21 ± 0.13 days. n = 3) (Fig. 4a,b). Then, we knocked out the gene *crc* in the strains *ΔcbrA* and *ΔcbrB* to generate the double-knockout strains *ΔcbrAΔcrc* and *ΔcbrBΔcrc*, respectively, which were assayed in the slow-killing experiment. As shown in Fig. 4a,b, deletion of *crc* in the strains *ΔcbrA* and *ΔcbrB* restored the virulence of these bacterial strains (the LT_50_ values were 3.23 ± 0.08 and 3.14 ± 0.08 days, respectively. n = 3). When re-expressing the *crc* gene in the double-knockout strains (*ΔcbrAΔcrc* and *ΔcbrBΔcrc*), the virulence of the bacteria was reduced (the LT_50_ values increased to 5.61 ± 0.15 and 5.31 ± 0.08 days, respectively. n = 3). These results reveal that in *P*. *donghuensis* HYS, the gene *crc* is essential for bacterial virulence via the CbrA/CbrB/CrcZ/CrcY system. Moreover, to further test the function of *crc*, we constructed an expression plasmid carrying *crc* and transformed it into the wild-type strain. As shown in Fig. 4c,d, overexpression of *crc* resulted in reduced virulence compared to that of the wild-type strain (the LT_50_ value increased from 3.17 ± 0.07 days to 5.91 ± 0.16 days. n = 3). These results show that the gene *crc* negatively regulates the virulence of *P*. *donghuensis* HYS.

### Identification of the correlation between screened virulence-related genes and Cbr/Crc system

After confirming the function of *crc*, we tried to find the target virulence factors directly modulated by Crc. As reported previously, virulence factors modulated by Crc in *Pseudomonas aeruginosa* can be categorized into quorum sensing system, secretion system, and single virulence factors^22–26^. After excluding the ones that do not exist in HYS, we analyzed the rest virulence factors in our experimental system on the level of transcriptional quantity. However, they all had small changes in the mutants (GEO Series accession number GSE108703). We also picked several genes to knock out in HYS, and test no virulence reduction (data not shown).

The transcriptomic analysis conducted in the strains *ΔcbrA*, *ΔcbrB*, and *ΔcrcYΔcrcZ* as well as the *crc*-overexpression strain was also utilized to investigate the correlations between the Cbr/Crc system and the remaining 66 previously screened virulence-related genes. As shown in Supplementary Table S4, 48 genes showed increased expression, suggesting negative regulation by the Cbr/Crc system, while the other 18 genes showed decreased expression, indicating positive regulation by this system. We also conducted Gene Ontology (GO) functional enrichment and Kyoto Encyclopedia of Genes and Genomes (KEGG) pathway enrichment analyses.

As shown in Supplementary Fig. S4a,c, the genes showing increased or decreased expression could be categorized into biological process, cellular component, and molecular function. These three categories contained 10, 4, and 5 functional groups of upregulated genes and 7, 5, and 7 functional groups of downregulated genes, respectively. Although different in number, the main functional groups for each category were similar in type between upregulated and downregulated genes. The functional categories were cellular process, single-organism process, metabolic process, catalytic activity, and binding. KEGG pathway analysis of the genes in each class showed that the main enriched pathways were metabolic pathways, two-component systems, biosynthesis of antibiotics, carbon metabolism, biosynthesis of secondary metabolites, and microbial metabolism in diverse environments (Supplementary Fig. S4b,d).

These results show that the remaining 66 screened virulence-related genes clustered in specific functional groups and biological pathways, although they were affected by different members of the Cbr/Crc system. Since the enriched functional groups and pathways were related to bacterial virulence, the Cbr/Crc system showed a chief regulatory function among these genes.

### Cbr/Crc-regulated virulence is related to *C*. *elegans* innate immunity

**Figure 5:**
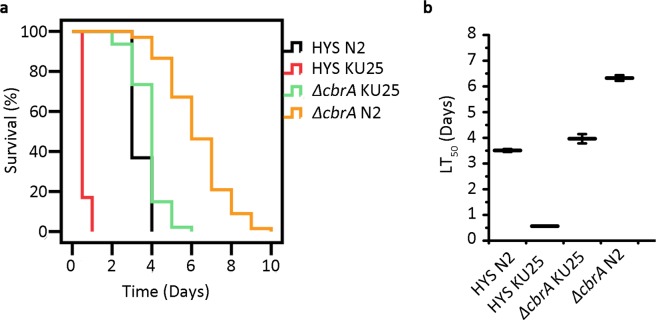
Correlation between Cbr/Crc-regulated virulence and worm innate immunity. (**a**,**b**) N2 and mutant worms deficient in the PMK-1/p38 MAPK pathway were fed *P*. *donghuensis* HYS and *ΔcbrA*, respectively. Curves are representative of three independent experiments. Data are presented as the mean ± standard deviation from three independent experiments.

To investigate the worm response to *P*. *donghuensis* HYS virulence, we utilized the relevant mutant KU25, which is deficient in the PMK-1/p38 MAPK pathway, to conduct a slow-killing assay. As shown in Fig. 5, after feeding on *P*. *donghuensis* HYS, mutant *C*. *elegans* worms died quickly, within one day. However, their viability improved markedly when we used the *cbrA* deletion mutant as an alternative food source, and their life span increased to six days, illustrating that as a food source for mutant worms, the strain *ΔcbrA* had lower virulence than the wild-type strain. The LT_50_ value of mutant worms fed *P*. *donghuensis* HYS was approximately 83.76% lower than that of N2 worms fed *P*. *donghuensis* HYS (the LT_50_ value decreased from 3.51 ± 0.06 days to 0.57 ± 0.02 days. n = 3). In addition, the LT_50_ value decreased by approximately 37.28% when the *cbrA* deletion mutant was provided as an alternative food source (the LT_50_ value decreased from 6.33 ± 0.11 days to 3.97 ± 0.18 days. n = 3). The difference between these two percentages suggests a correlation between PMK-1/p38 MAPK innate immunity in *C*. *elegans* and *cbrA*-related virulence of *P*. *donghuensis* HYS.

## Discussion

**Figure 6:**
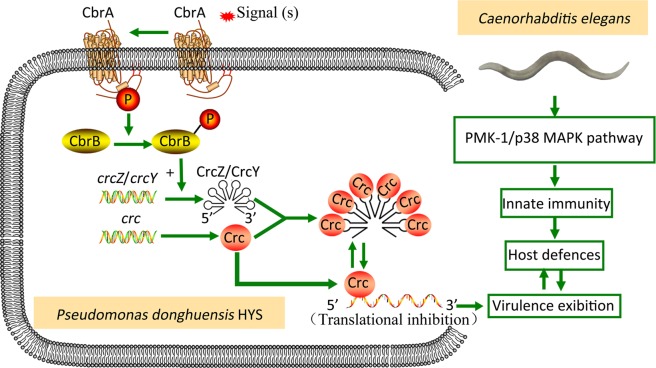
Proposed regulation of the Cbr/Crc system in a *P*. *donghuensis* HYS-*C*. *elegans* slow-killing assay. Under normal conditions, CbrA functions through CbrB to promote the transcription of *crcZ* and *crcY*. The small RNAs CrcZ and CrcY bind to the Crc protein to inhibit its binding to virulence-related genes. Thus, the *P*. *donghuensis* HYS strain exhibits virulence characteristics. When encountering *C*. *elegans*, *P*. *donghuensis* HYS virulence may trigger a host response through the PMK-1/p38 MAPK innate immune pathway.

The Cbr/Crc system functions to regulated the virulence of *P*. *donghuensis* HYS through negative regulation by Crc. Deficiency of *crc* in the wild-type strain did not alter the worm life span. However, *crc* deletion led to increased virulence in *ΔcbrA* and *ΔcbrB* strains (Fig. 4a,b), and overexpression of *crc* led to decreased virulence compared with that of the wild-type strain (Fig. 4c,d). These results are different from those of previous studies, in which *crc* functioned positively in almost all reported model systems, including human epithelial cell lines, *Dictyostelium discoideum*, lettuce, mouse lungs, and the tomato and *Arabidopsis* dip-inoculation model systems, with only one report describing a weak negative correlation between *crc* expression and virulence in *P*. *aeruginosa*^11,20,23,24,26,27^. Crc is the major regulator of the Cbr/Crc system, and the other components (CbrA, CbrB, CrcZ, and CrcY) in this study functioned in the same patterns as previously reported. Thus, the different phenotypes of the corresponding mutants depended on the different functions of Crc. A possible virulence mechanism of the Cbr/Crc regulatory system in *P*. *donghuensis* HYS is proposed in Fig. 6, which may depict the mechanism of virulence regulation in the whole species.

The variations in bacterial virulence in this study may be due to the direct binding of Crc to target mRNAs corresponding to virulence-related genes. According to its protein structure, Crc likely binds to the A-rich motif located in the 5’region of a target mRNA and inhibits its translation^21,28^. Under normal conditions, CrcZ and CrcY competitively bind to Crc. Thus, the mRNAs of virulence genes are released, and virulence is exhibited in *P*. *donghuensis* HYS. However, when Crc is no longer bound to these small RNAs or when excess Crc is present in the cell, the translation of the target mRNAs is inhibited, causing a reduced virulence phenotype. As for the target genes for Crc binding, we have conducted several tests. Among the previously reported virulence factors, the Rhl and PQS QS systems reported being modulated by Crc do not exist in HYS, and the rest are not significant according to transcriptional analysis. Among the non significant genes,we tested the T6SS and *hcnB* gene. Results show that deletion of the T6SS or *hcnB* did not affect the virulence of HYS. In conclusion, we are still working on finding the target virulence genes which Crc may modulate in our experimental system since the previously reported virulence factors under the modulation of Crc are all not applicable in our novel Cbr/Crc regulatory system. The mechanism underlying the previously reported results may be this indirect binding between Crc and virulence-gene mRNAs. For example, the expression of T3SS virulence genes is regulated by *crc* through *ptrB*, *cpdA*, *rpoS*, and *prpC* expression^24^.

The PMK-1/p38 MAPK pathway is the most crucial pathway in intestinal innate immunity^29^ and was suitable for investigating the correlation between pathogenicity and host response in this study. ZG31 *hif-1* (ia4) worms^30,31^ were used in this paradigm to eliminate the background susceptibility of mutant worms (Supplementary Fig. S5). The results showed a response to *cbrA*-regulated virulence of *P*. *donghuensis* HYS mediated by the PMK-1/p38 MAPK pathway, which has not been reported previously (Fig. 6). This pathway may supplement the virulence mechanism based on the Cbr/Crc regulatory system.

Deletion of the *cbrA* gene does not abolish virulence in *P*. *donghuensis* HYS (Fig. 3b), indicating that other virulence factors exist in this bacterium. Pyoverdine, which has a high affinity to iron, is the major chelating compound secreted by *P*. *donghuensis* HYS and is required for pathogenesis in *C*. *elegans*^15,32,33^. As shown in Supplementary Fig. S6, the LT_50_ value of the *pvdA* deletion mutant was 5.53 ± 0.17 days, approximately 1.74-times higher than that of *C*. *elegans* fed the wild-type strain, showing that pyoverdine contributes to virulence in *P*. *donghuensis* HYS.

In summary, we found that the Cbr/Crc system regulates the virulence of *P*. *donghuensis* HYS in a *C*. *elegans* slow-killing assay through a novel functional mechanism in which Crc has a negative effect and that the virulence of this bacterium is related to the PMK-1/p38 MAPK pathway in *C*. *elegans*.

## Methods

### Bacteria and nematodes and ethics statement

The bacterial strains and plasmids used in this study are listed in Supplementary Table S5. The *Escherichia coli* strains were grown in Luria-Bertani (LB) broth at 37 °C. *P*. *donghuensis* strains were grown in LB broth at 30 °C. When necessary, antibiotics were added at the following final concentrations: for *P*. *donghuensis* strains, 25 μg/ml chloramphenicol, 50 μg/ml kanamycin, and 50 μg/ml gentamicin; for *E*. *coli* strains, 50 μg/ml kanamycin and 10 μg/ml gentamicin.

The *C*. *elegans* wild-type Bristol N2, KU25 *pmk-1*(km25) and ZG31 *hif-1*(ia4) strains (*Caenorhabditis* Genetics Center) were used in this study. For routine maintenance, overnight cultures of *E*. *coli* OP50^34^ were spread on nematode growth medium (NGM) agar plates and then incubated for 8 hours at 37 °C as the food source. To obtain synchronous day-1 adult worms, the eggs laid over half an hour were collected and grown at 22 °C. Worm stocks were subjected to bleach treatment^35^ to remove contaminants, and worms from the generation after bleaching were used for the experiments. Ethic approval for using *C*. *elegans* in this study was not necessary because *C*. *elegans* was not covered by any ethical committee.

### Food-avoidance assay

For the food-avoidance assay, overnight cultures of the tested strains were dropped on NGM plates. After incubation at 22 °C for 12 hours, 50 adult worms were placed in the centre of the bacterial lawn. The ratio of worms on the lawn versus total worms on the plate was calculated every 2 hours. The experiment was performed three times independently.

### Slow-killing assay

The slow-killing assays were performed by spreading 150 μl of overnight culture (*OD*_600_ is approximately 3.5) of the tested bacterial strain onto NGM agar plates. Then, the plates were incubated for 12 hours at 22 °C. A total of 100 adult worms for each tested strain were transferred to plates containing pre-grown bacterial lawns, and then the plates were incubated at 22 °C. To eliminate the effect of generation, transfer of the worms to new plates with pre-grown bacterial lawns was conducted every day until the end of the experiment. Worms were scored daily under an SZM-45B1 stereomicroscope (Sunny Optical, Yuyao, China), and those that did not respond to touch with a platinum wire picker were considered dead, while those that ruptured, bagged or crawled up the sides of the plates and dried out were censored^36^. The LT_50_ value was taken from Kaplan-Meier survival curves and was used to evaluate bacterial virulence. This experiment was performed three times independently,

### Choice assay

Preference for bacterial strain was analysed using a standard choice assay^37^ with modifications. First, 90-mm NGM plates were seeded with cultures of *P*. *donghuensis* HYS and *E*. *coli* OP50 at locations on opposite sides of the centre point. Bacterial cultures were equidistant from the centre point. After 12 hours of incubation at 22 °C, 50 adult worms were moved to a drop of M9 buffer at the centre of the plate. The number of worms on each bacterial lawn was counted after 6 hours, and the choice index was calculated as (number of worms on the tested bacterial lawn-number of worms on the *E*. *coli* OP50 lawn)/total number of worms. This experiment was performed ten times independently.

### Transposon mutagenesis and screening for mutants with reduced ability to repel worms

The minitransposon vector pBT20 was introduced into *P*. *donghuensis* HYS by means of bacterial conjugation to generate insertion mutants as previously described^38^. Then, the potential virulence-reduced mutants were screened according to the nematode preference for bacterial strains^19^. First, preliminary screening was carried out. The tested mutants were inoculated separately into 96-well plates containing LB broth supplemented with gentamicin and chloramphenicol in each well. After incubation with shaking at 30 °C, 10 μl of each mutant culture was dropped onto NGM plates. Four drops of *E*. *coli* OP50 culture were placed at opposite sides of the mutant grid as a food source. The plates were then incubated at 22 °C for 12 hours to allow bacterial growth, and then, approximately 50 adult worms were placed adjacent to the *E*. *coli* OP50 lawns. The consumption of the lawns was observed after 3 days. The transposon mutants were considered repellence-reduced when the corresponding lawns on the NGM plates were mostly consumed. Mutants passing this preliminary screening were then streaked onto LB plates supplemented with gentamicin and chloramphenicol to obtain single colonies for the second screening, which was performed as described for the preliminary screening, except that the mutant and wild-type cultures were dropped in separate lines, with each line of mutant culture placed between two lines of wild-type culture.

### Determination of insertion sites

The DNA segments adjacent to the transposon insertions were obtained using TAIL-PCR and arbitrarily primed PCR, as previously described^39,40^. U1, U2, and U3 were nested sequence-specific primers, and the arbitrary degenerate primer was selected from AD-1, AD-2, AD-3, AD-4, and AD-5^15^. The products were purified from agarose gel and sequenced using the primer U3. Then, each sequence was aligned to the HYS whole-genome shotgun contigs in the National Center for Biotechnology Information (NCBI) database to identify affected genes^41^. The corresponding proteins were analysed online at NCBI (http://www.ncbi.nlm.nih.gov/Structure/cdd/wrpsb.cgi) and SMART (http://smart.embl-heidelberg.de/), and the pathways that they may affect were analysed at KEGG (http://www.kegg.jp/kegg/).

### DNA manipulation and plasmid construction

The primers used in this study are listed in Supplementary Table S6. Routine genetic manipulation, including PCR, agarose gel electrophoresis, restriction enzyme digestion, and transformation, was performed using standard procedures^42^. Chromosomal DNA from *P*. *donghuensis* HYS was extracted with a Genomic DNA Purification Kit (Promega, Madison, WI, USA). Plasmid DNA was isolated with a Plasmid Mini Kit I (Omega Bio-Tek, Norcross, GA, USA). Agarose gel fragments were purified using a Gel Extraction Kit (Omega Bio-Tek, Norcross, GA, USA). All restriction endonucleases were purchased from Thermo Fisher Scientific (Waltham, MA, USA). Primer synthesis and DNA sequencing were carried out by Sangon Biotech (Shanghai, China).

Complementation and overexpression plasmids were constructed by ligating the Shine-Dalgarno sequences and open reading frames (ORFs) of the target genes into pBBR1MCS-2^43^ (pBBR2 for short).

### Construction of in-frame deletion mutants

Primers were designed to amplify fragments located upstream and downstream of each target gene. After digestion with specific enzymes, the two amplified fragments were ligated into the suicide vector pEX18Gm^44^. After sequencing, the correct recombinant plasmid was transformed into *P*. *donghuensis* HYS via conjugation from *E*. *coli* S17-1 λ*pir*^45^. The target gene was knocked out by allelic exchange, and selection for double recombinants was performed on LB agar plates containing 5% (wt/vol) sucrose. The correct gene deletion mutants were further confirmed by PCR and sequencing.

### Prediction of sRNA secondary structure

Secondary structures of sRNAs were predicted using the RNAfold algorithm available at the ViennaRNA Web Services (http://rna.tbi.univie.ac.at/).

### Growth curve analysis

The wild-type and mutant *P*. *donghuensis* strains were grown in LB broth at 30 °C until the stationary phase. Then, they were transferred to fresh LB broth in a ratio of 1:100 (vol/vol) separately. The cultures were incubated at 30 °C with shaking, and bacterial growth was monitored every 2 hours for 30 hours. For each time point, a V-1200 spectrophotometer (Mapada, Shanghai, China) was used to determine the optical density of the bacterial cultures at 600 nm (*OD*_600_). This experiment was performed three times independently.

### RNA-Seq library construction, sequencing, and data analysis

Transcriptomic analyses of *ΔcbrA*, *ΔcbrB* and *ΔcrcZΔcrcY* strains were performed with *P*. *donghuensis* HYS as the control. Transcriptomic analysis of *P*. *donghuensis* HYS/pBBR2-*crc* was performed with *P*. *donghuensis* HYS/pBBR2 as the control. The sequences were processed and analysed by BGI (Shenzhen, China). In addition, genes were aligned against several databases, including the NCBI nonredundant protein database (http://www.ncbi.nlm.nih.gov) and the KEGG pathway database (http://www.genome.jp/kegg) by BLASTX.

### GO analysis and KEGG pathway enrichment analysis

To investigate their biological functions, target genes were first annotated in the GO database (http://www.geneontology.org/) and then classified into relative functional classes. *P*-values were subjected to Bonferroni correction with a corrected *P*-value ≤ 0.05. Meanwhile, KEGG pathway enrichment analysis was performed utilizing the KEGG pathway database (http://www.genome.jp/kegg).

### Statistical analysis

All data are presented as the mean ± standard deviation, and each experiment was performed at least three times independently. Statistical analysis was performed by using IBM SPSS version 18.0 (SPSS Inc., Chicago, USA) and OriginPro 8.0 (OriginLab, USA). Survival curves were plotted with the Kaplan-Meier method using SPSS. Significant differences between the treatments were determined by Student’s t-test

### Microscopy

Experiments involving worms were performed by utilizing the SZM-45B1 stereomicroscope (Sunny Optical, Yuyao, China). Photographs were taken under the stereomicroscope with a Leica M240 digital camera (Leica, Germany).

### Accession numbers

The GenBank accession numbers for the proteins CbrA, CbrB, Crc, and PvdA from *P*. *donghuensis* HYS are WP_036995606.1, WP_010222999.1, WP_010225880.1, and WP_010222460, respectively. The sRNA-encoding genes *crcZ* and *crcY* are located at NZ_JH650764.1 (198431–198825) and NZ_JH650785.1 (1586–1978), respectively.

Next-generation sequencing data accession numbers: The transcriptomic data described in this study have been deposited in the NCBI Gene Expression Omnibus^46^ and are accessible through GEO Series accession number GSE108703 (https://www.ncbi.nlm.nih.gov/geo/query/acc.cgi?acc=GSE108703).

## Supplementary information

Pseudomonas donghuensis HYS virulence towards Caenorhabditis elegans is regulated by the Cbr/Crc system

## Data Availability

All data generated or analysed during this study are included in this published article (and its Supplementary Information files).

## References

[1] Hornef MW, Wick MJ, Rhen M, Normark S (2002). Bacterial strategies for overcoming host innate and adaptive immune responses. Nature immunology.

[2] Rohmer L, Hocquet D, Miller SI (2011). Are pathogenic bacteria just looking for food? Metabolism and microbial pathogenesis. Trends in microbiology.

[3] Shen S, Fang FC (2012). Integrated stress responses in *Salmonella*. International journal of food microbiology.

[4] Duprey A, Reverchon S, Nasser W (2014). Bacterial virulence and Fis: adapting regulatory networks to the host environment. Trends in microbiology.

[5] Wang HR, Hu YH, Zhang WW, Sun L (2009). Construction of an attenuated *Pseudomonas fluorescens* strain and evaluation of its potential as a cross-protective vaccine. Vaccine.

[6] Xin XF, He SY (2013). *Pseudomonas syringae* pv. tomato DC3000: a model pathogen for probing disease susceptibility and hormone signaling in plants. Annual review of phytopathology.

[7] Fernandez M (2015). Analysis of the pathogenic potential of nosocomial *Pseudomonas putida* strains. Frontiers in microbiology.

[8] Al-Wrafy F, Brzozowska E, Gorska S, Gamian A (2017). Pathogenic factors of *Pseudomonas aeruginosa* - the role of biofilm in pathogenicity and as a target for phage therapy. Postepy higieny i medycyny doswiadczalnej.

[9] Francis, V. I., Stevenson, E. C. & Porter, S. L. Two-component systems required for virulence in *Pseudomonas aeruginosa*. *FEMS microbiology letters* **364**, 10.1093/femsle/fnx104 (2017). 10.1093/femsle/fnx104 PMC5812489 28510688

[10] Klockgether J, Tummler B (2017). Recent advances in understanding *Pseudomonas aeruginosa* as a pathogen. F1000Research.

[11] Yeung ATY, Bains M, Hancock REW (2011). The Sensor Kinase CbrA Is a Global Regulator That Modulates Metabolism, Virulence, and Antibiotic Resistance in *Pseudomonas aeruginosa*. Journal of bacteriology.

[12] Gao J, Xie G, Peng F, Xie Z (2015). *Pseudomonas donghuensis* sp. nov., exhibiting high-yields of siderophore. Antonie van Leeuwenhoek.

[13] Ossowicki A, Jafra S, Garbeva P (2017). The antimicrobial volatile power of the rhizospheric isolate *Pseudomonas donghuensis* P482. PloS one.

[14] Agaras BC, Iriarte A, Valverde CF (2018). Genomic insights into the broad antifungal activity, plant-probiotic properties, and their regulation, in *Pseudomonas donghuensis* strain SVBP6. PloS one.

[15] Yu XY, Chen M, Jiang Z, Hu Y, Xie ZX (2014). The Two-Component Regulators GacS and GacA Positively Regulate a Nonfluorescent Siderophore through the Gac/Rsm Signaling Cascade in High-Siderophore-Yielding *Pseudomonas* sp Strain HYS. Journal of bacteriology.

[16] Sorathia N, Rajadhyaksha MS (2016). *Caenorhabditis elegans*: A Model for Studying Human Pathogen Biology. Recent patents on biotechnology.

[17] Dzvova N, Colmer-Hamood JA, Griswold JA, Hamood AN (2017). Isolation and characterization of HepP: a virulence-related *Pseudomonas aeruginosa* heparinase. BMC microbiology.

[18] van Tilburg Bernardes, E., Charron-Mazenod, L., Reading, D. J., Reckseidler-Zenteno, S. L. & Lewenza, S. Exopolysaccharide-Repressing Small Molecules with Antibiofilm and Antivirulence Activity against *Pseudomonas aeruginosa*. *Antimicrobial agents and chemotherapy* **61**, 10.1128/AAC.01997-16 (2017). 10.1128/AAC.01997-16 PMC5404518 28223377

[19] Lewenza Shawn, Charron-Mazenod Laetitia, Giroux Lauriane, Zamponi Alexandra D. (2014). Feeding behaviour ofCaenorhabditis elegansis an indicator ofPseudomonas aeruginosaPAO1 virulence. PeerJ.

[20] Linares JF (2010). The global regulator Crc modulates metabolism, susceptibility to antibiotics and virulence in *Pseudomonas aeruginosa*. Environmental microbiology.

[21] Sonnleitner E, Abdou L, Haas D (2009). Small RNA as global regulator of carbon catabolite repression in *Pseudomonas aeruginosa*. Proceedings of the National Academy of Sciences of the United States of America.

[22] Yang N (2015). The Crc protein participates in down-regulation of the Lon gene to promote rhamnolipid production and *rhl* quorum sensing in *Pseudomonas aeruginosa*. Molecular microbiology.

[23] Zhang L (2013). Regulation of *pqs* quorum sensing via catabolite repression control in *Pseudomonas aeruginosa*. Microbiology.

[24] Dong YH, Zhang XF, Zhang LH (2013). The global regulator Crc plays a multifaceted role in modulation of type III secretion system in *Pseudomonas aeruginosa*. MicrobiologyOpen.

[25] Browne P, Barret M, O’Gara F, Morrissey JP (2010). Computational prediction of the Crc regulon identifies genus-wide and species-specific targets of catabolite repression control in *Pseudomonas* bacteria. BMC microbiology.

[26] Janjua HA (2012). Clinical populations of *Pseudomonas aeruginosa* isolated from acute infections show a wide virulence range partially correlated with population structure and virulence gene expression. Microbiology.

[27] Chakravarthy S (2017). Virulence of *Pseudomonas syringae* pv. tomato DC3000 Is Influenced by the Catabolite Repression Control Protein Crc. Molecular plant-microbe interactions: MPMI.

[28] Moreno R, Marzi S, Romby P, Rojo F (2009). The Crc global regulator binds to an unpaired A-rich motif at the *Pseudomonas putida alkS* mRNA coding sequence and inhibits translation initiation. Nucleic acids research.

[29] Pukkila-Worley R, Ausubel FM (2012). Immune defense mechanisms in the *Caenorhabditis elegans* intestinal epithelium. Curr Opin Immunol.

[30] Jiang H, Guo R, Powell-Coffman JA (2001). The *Caenorhabditis elegans hif-1* gene encodes a bHLH-PAS protein that is required for adaptation to hypoxia. Proceedings of the National Academy of Sciences of the United States of America.

[31] Nystul TG, Roth MB (2004). Carbon monoxide-induced suspended animation protects against hypoxic damage in *Caenorhabditis elegans*. Proceedings of the National Academy of Sciences of the United States of America.

[32] Kirienko NV (2013). *Pseudomonas aeruginosa* disrupts *Caenorhabditis elegans* iron homeostasis, causing a hypoxic response and death. Cell host & microbe.

[33] Kirienko NV, Ausubel FM, Ruvkun G (2015). Mitophagy confers resistance to siderophore-mediated killing by *Pseudomonas aeruginosa*. Proceedings of the National Academy of Sciences of the United States of America.

[34] Brenner S (1974). The genetics of *Caenorhabditis elegans*. Genetics.

[35] Stiernagle, T. Maintenance of *C*. *elegans*. *WormBook: the online review of C*. *elegans biology*, 1–11; 10.1895/wormbook.1.101.1 (2006). 10.1895/wormbook.1.101.1 PMC4781397 18050451

[36] Hansen M (2007). Lifespan extension by conditions that inhibit translation in *Caenorhabditis elegans*. Aging cell.

[37] Abada EA (2009). *C*. *elegans* behavior of preference choice on bacterial food. Molecules and cells.

[38] Kulasekara HD (2005). A novel two-component system controls the expression of *Pseudomonas aeruginosa* fimbrial *cup* genes. Molecular microbiology.

[39] Liu YG, Whittier RF (1995). Thermal asymmetric interlaced PCR: automatable amplification and sequencing of insert end fragments from P1 and YAC clones for chromosome walking. Genomics.

[40] Das S, Noe JC, Paik S, Kitten T (2005). An improved arbitrary primed PCR method for rapid characterization of transposon insertion sites. Journal of microbiological methods.

[41] Gao J, Yu X, Xie Z (2012). Draft genome sequence of high-siderophore-yielding *Pseudomonas* sp. strain HYS. Journal of bacteriology.

[42] Chong L (2001). Molecular cloning - A laboratory manual, 3rd edition. Science.

[43] Kovach ME (1995). Four new derivatives of the broad-host-range cloning vector pBBR1MCS, carrying different antibiotic-resistance cassettes. Gene.

[44] Hoang TT, Karkhoff-Schweizer RR, Kutchma AJ, Schweizer HP (1998). A broad-host-range Flp-FRT recombination system for site-specific excision of chromosomally-located DNA sequences: application for isolation of unmarked *Pseudomonas aeruginosa* mutants. Gene.

[45] Simon R, Priefer U, Puhler A (1983). A Broad Host Range Mobilization System for Invivo Genetic-Engineering - Transposon Mutagenesis in Gram-Negative Bacteria. Bio-Technol.

[46] Edgar R, Domrachev M, Lash AE (2002). Gene Expression Omnibus: NCBI gene expression and hybridization array data repository. Nucleic acids research.

