# Supplementary material for: Pseudomonas donghuensis HYS virulence towards Caenorhabditis elegans is regulated by the Cbr/Crc system
Source: Sci Rep. 2019 Jun 19;9:8772. doi: 10.1038/s41598-019-45145-8 (PMC6584532; doi:10.1038/s41598-019-45145-8)
Supplement: Supplementary file 1 — Pseudomonas donghuensis HYS virulence towards Caenorhabditis elegans is regulated by the Cbr/Crc system [file 41598_2019_45145_MOESM1_ESM.pdf]

Supplementary Material

Pseudomonas donghuensis HYS virulence towards *Caenorhabditis elegans* is regulated by the Cbr/Crc system

Guanfang Xie, Man Zeng, Jia You, Zhixiong Xie*

***Correspondence:** Zhixiong Xie: zxxie@whu.edu.cn

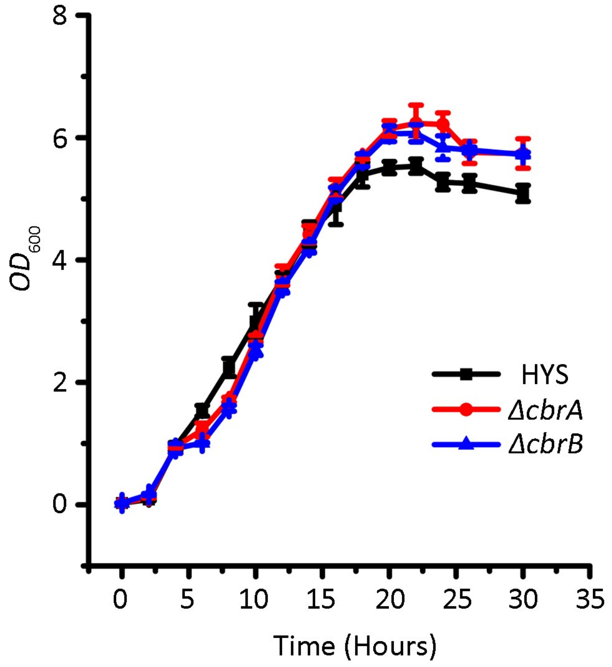

Supplementary Figure S1. Growth of the *P. donghuensis* HYS, *ΔcbrA*, and *ΔcbrB* strains. Data are presented as the mean ± standard deviation from three independent experiments.

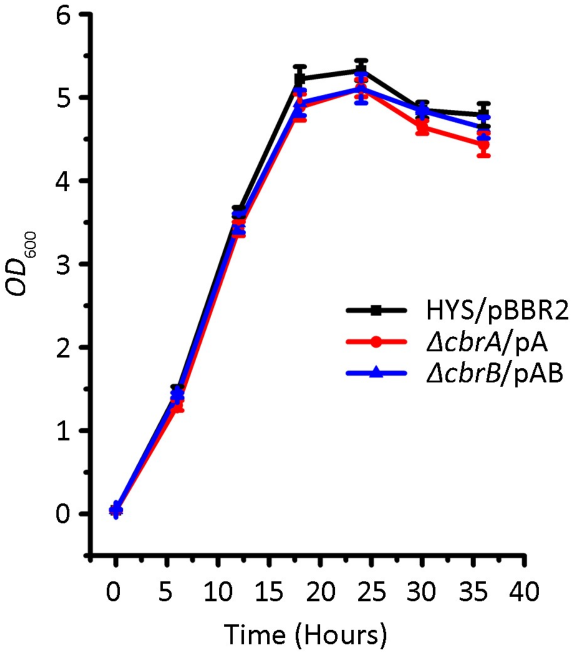

Supplementary Figure S2. Growth of the *P. donghuensis* HYS/pBBR2, *ΔcbrA*/pA, and *ΔcbrB*/pAB strains. Data are presented as the mean ± standard deviation from three independent experiments.

a

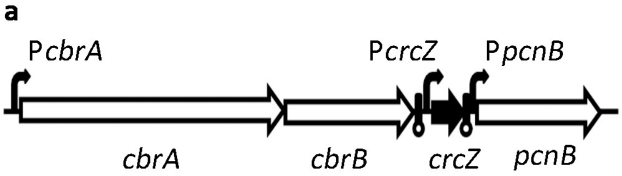

cbrB (end)

TGAGCCGTTTCAGCGCTTTGCGCGGGTGGGCACAGGTAACACCG
CTACCTTGCGACAAACTGTTACCGCACCACAAAGTCGTAACAAAA
ACCGGGGCTTACGGTAACGAAGCCGCGGTTTTTTTTCGCCTTGCG
AACCGCTCAAAACCGCTCCACCCCCTTGTTTTACTGGGTTTTGAAA
AAGTTGGCACGGCACCTGCTATATGTTTGGTACAAGAACAATAAC
AAGCACTGCAAAAGACAATAAAAATAAGACGAATCGGCTCACGC
ACAATAAGAACAAGACGGCGGAGGCGCAGCTAACTGATTCTTTT
GGAGAGGAGTTGTATTTGGGGCTTGCCCCACAACCAGGCAGAGA
ACAATAAAAACTGCACTCAAGCAGGGCCTGAACTGGTTGGATCG
CATGATCATTGCAACCTCAGCGACCAAAGCAATCCGTTTGCTCTT
GATCCCGATTAGGGAGACCGCCCACAAGCTTTTTTTGTGGGCAG
GGCACTCAACAAAAACAAAAAGCCCGGCAAAAAAACAATAAGAG
CACGCAACTACTTCTTGGGGAGCTTCGGCTCCCCTTGTAGTTTCTG

CCCGCCCCAGCCCGCCTGTGCGTCGTCCGACGCGACCGCCTACAC
CATCCTCCGAGTAAATGCTAGAATCCCCGCCCATCATGCGGCCAT
TCTTCGTTTTTGGCCGAACATTCCTTCAAACAGTGCATCCCATG

pcnB →

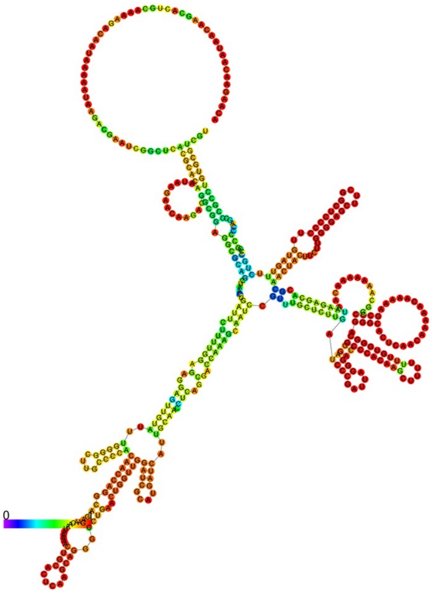

b

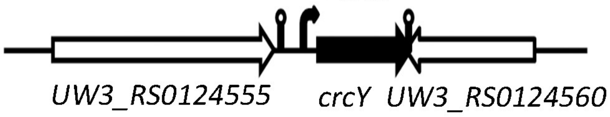

UW3-RS0124555 (end)

TGAACACAGGCGGTAACAGCCCAATCACCCCCTGGGTGT
TACCGCGCCCGCACTTTTCAGGGAAATTCCGGGTAACAC
GGAAACAAATCGACAGAATTTCCCGGGACCGTTTTTTGAT
AAAACCTTCAAACCATTGATTTTATTGGGTTTTAAAAAGTT
GGCACGGCACCTGCTATATCTCAGGTACAAGAACAATAA
AAAGTGCGACATACCCAATAAAAATAAGACGAAACGACT
CTGACATAACAAGAACAACACGACAGAGGCGCAGCTAAC
AGATTTTTTTGGAGAGGATGTGCTTTCCAGGGGCTTGCCC
CGCAACCGGACACAAAACAATAAAACTACCCCAAGGTAG
CGCAGGTCACGGTTGGATCAGCAATGATGAAGGTAGATC
AGCGCTCAAAAAAATACGTTTGCTCTTGATCCCGGATGG
GGATCGCCAGTGATGCAGAGGCAACGGTCACCAAAAACA
ACAATAGGCCGCCCTCCAATAACAAAAATGAGAGCACGC
AACAATCTTTGAGGGGAGCTTAGGCTCCCCTCAGTGCTTC

UW3-RS0124555 (end)

CCGCCCTCGCCGTTTTCGTCCTCGTCCTTCTCAAAGCGATT
GACCACCAACGGCA

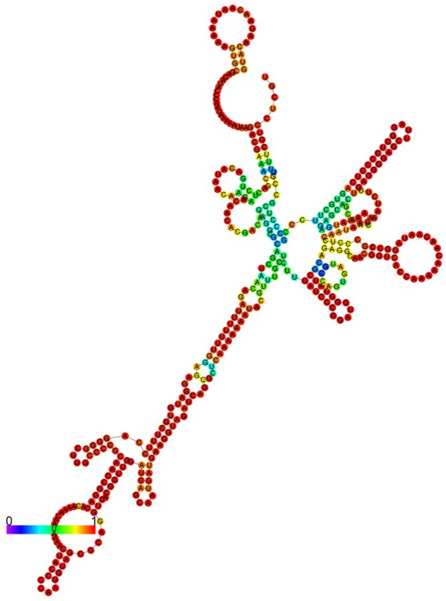

Supplementary Figure S3. Sequences, gene contexts and predicted secondary structures of the *P. donghuensis* HYS sRNAs, (**a**) CrcZ and (**b**) CrcY. The nucleotide sequences of *crcZ* and *crcY* are indicated in bold. Sequences encoding Crc binding sites are underlined and shown in red.

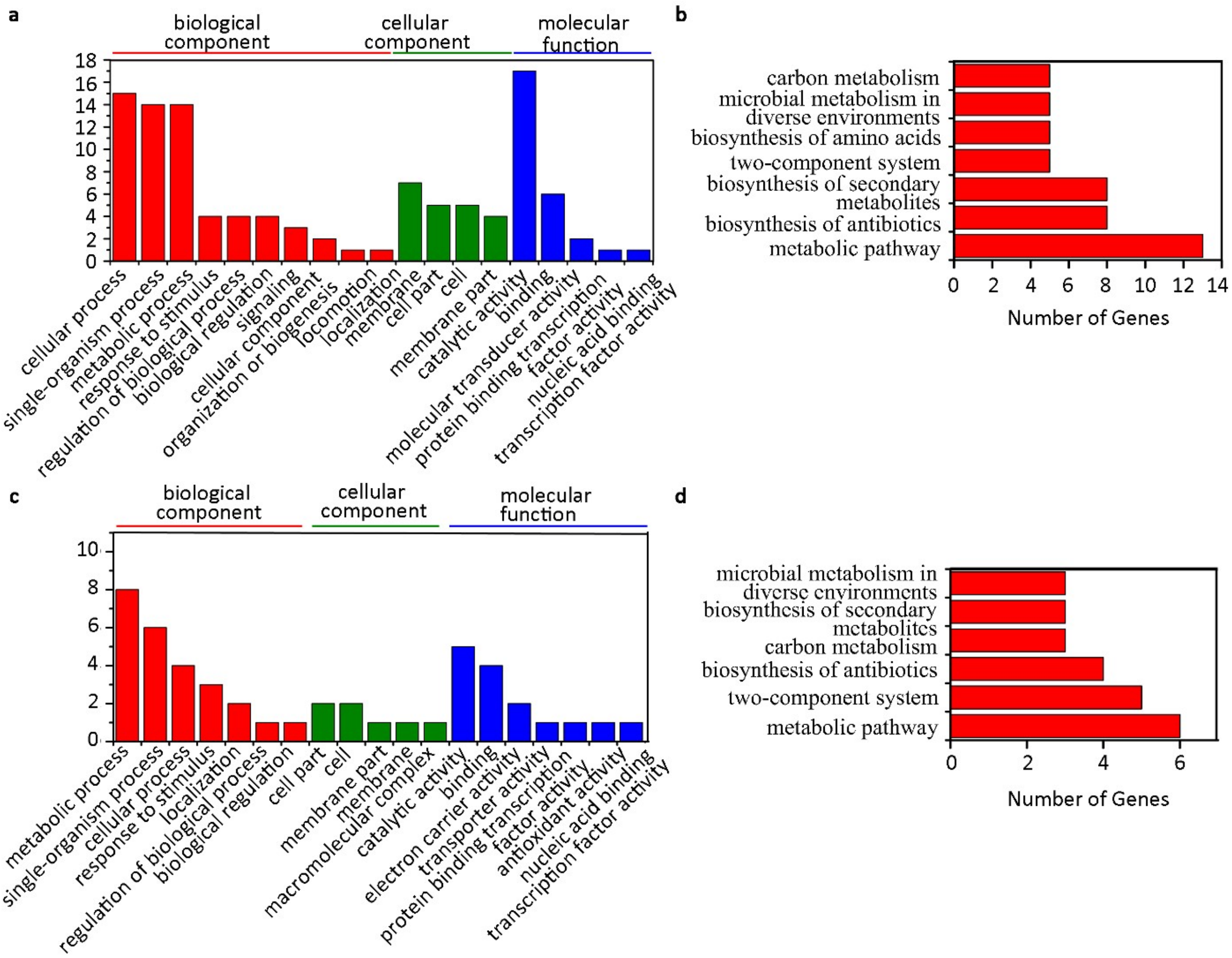

Supplementary Figure S4. GO functional enrichment and KEGG pathway enrichment analyses of the remaining 66 screened genes based on the correlations between their expression levels and Cbr/Crc regulation. (**a, b**) Forty-eight genes exhibiting higher expression in the treatment strain than in *P. donghuensis* HYS or *P. donghuensis* HYS/p were classified. (**c, d**) Eighteen genes with lower expression in the treatment strain than in *P. donghuensis* HYS or *P. donghuensis* HYS/p were classified.

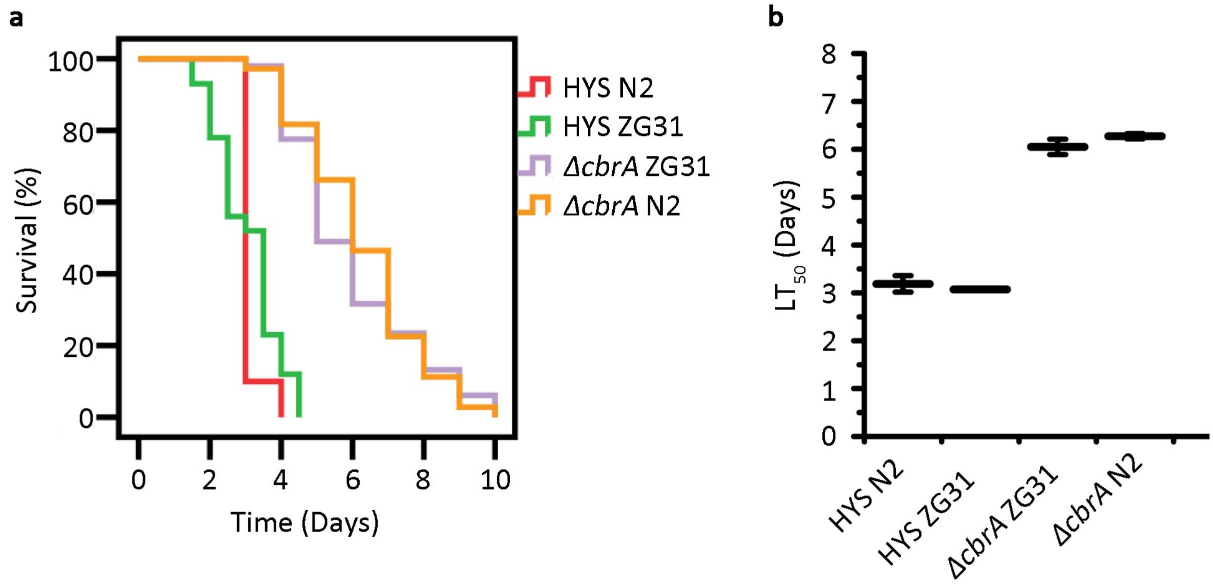

Supplementary Figure S5. Elimination of *C. elegans* mutants' general sensitivity in the slow-killing assay. (**a, b**) N2 and hypoxic-response-deficient mutant worms ZG31 were fed with *P. donghuensis* HYS and *ΔcbrA* strains, respectively. Curves are representative of three independent experiments. Data are presented as the mean ± standard deviation from three independent experiments.

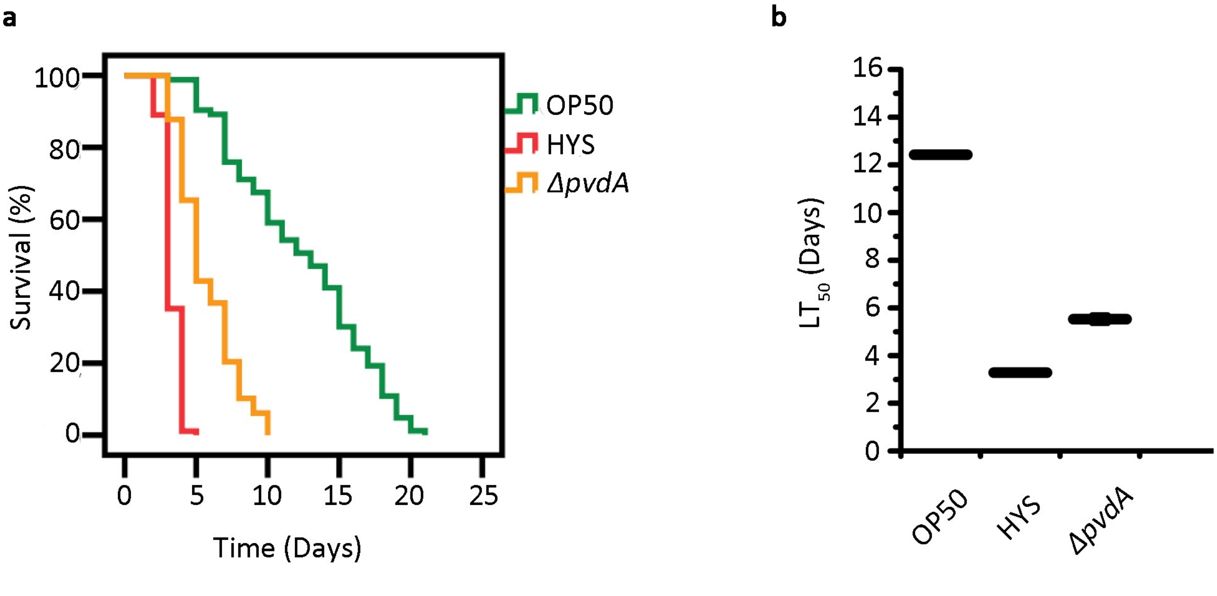

Supplementary Figure S6. Pyoverdine contributes to bacterial virulence in *P. donghuensis* HYS. (**a, b**) The effects of pyoverdine in virulence were assessed based on survival curves and LT_{50} values of worms fed a *pvdA* deletion mutant. Curves are representative of three independent experiments. Data are presented as the mean ± standard deviation from three independent experiments.

Supplementary Table S1. Transposon insertion sites of mutants identified by screening for reduced HYS repellence

Insertion site	Identity	Protein ID	Protein
Scaffold 1 38526-38527	UW3_RS0102520	WP_010220635.1	amino acid metabolite efflux pump
Scaffold 1 53875-53876	UW3_RS0102580	WP_010220648.1	PAS domain S-box protein
Scaffold 1 62596-62597	UW3_RS0102620	WP_010220659.1	TonB-dependent receptor
Scaffold 1 348413-348414	UW3_RS0103900	WP_010220971.1	RNA polymerase sigma factor RpoE
Scaffold 2 159889-159890	UW3_RS0105350	WP_010221262.1	outer membrane porin, OprD family
Scaffold 2 185441-185442	UW3_RS0105440	WP_026001200.1	type VI secretion system lipoprotein TssJ
Scaffold 2 204840-204841	UW3_RS0105515	WP_036995327.1	hypothetical protein
Scaffold 2 291005-291006	UW3_RS0105940	WP_010221378.1	pyruvate dehydrogenase (acetyl-transferring), homodimeric type
Scaffold 2 332202-332203	UW3_RS0106110	WP_026001208.1	MOSC domain-containing protein
Scaffold 2 346446-346447	UW3_RS0106175	WP_026001209.1	transcriptional regulator
Scaffold 3 35451-35452	UW3_RS0106600	WP_010221513.1	methylenetetrahydrofolate reductase [NAD(P)H]
Scaffold 3 118890-118891	UW3_RS0106950	WP_010221584.1	TIR domain-containing protein
Scaffold 3 128460-128461	UW3_RS0106995	WP_010221593.1	tRNA (adenosine(37)-N6)-dimethylallyl transferase MiaA
Scaffold 3 188651-188652	UW3_RS0107275	WP_010221650.1	urea ABC transporter ATP-binding protein UrtD
Scaffold 3 221405-221406	UW3_RS0107430	WP_010221683.1	tRNA dihydrouridine synthase DusB
Scaffold 3 270866-270867	UW3_RS0107620	WP_010221729.1	methyltransferase domain-containing protein
Scaffold 3 298275-298276	UW3_RS0107755	WP_010221756.1	methylmalonate-semialdehyde dehydrogenase (CoA acylating)
Scaffold 4 32850-32851	UW3_RS0107920	WP_010221794.1	CoA transferase subunit A
Scaffold 4 115279-115280	UW3_RS0108300	WP_010221877.1	two-component system response regulator UvrY
Scaffold 4 204936-204937	UW3_RS0108730	WP_010221972.1	protein-glutamate O-methyltransferase CheR
Scaffold 5 9638-9639	UW3_RS0109160	WP_010222066.1	ABC transporter substrate-binding protein
Scaffold 5 155604-155605	UW3_RS0109860	WP_010222233.1	cell division protein
Scaffold 5 186598-186599	UW3_RS0110010	WP_010222267.1	thioredoxin TrxC
Scaffold 6 236951-236952	UW3_RS0111395	WP_010222573.1	hypothetical protein
Scaffold 6 241825-241826	UW3_RS0111420	WP_010222576.1	phosphoesterase PHP domain-containing protein
Scaffold 7 69348-69349	UW3_RS0111745	WP_010222657.1	oligopeptidase A
Scaffold 7 150642-150643	UW3_RS0112125	WP_010222750.1	hypothetical protein

Insertion site	Identity	Protein ID	Protein
Scaffold 8 173223-173224	UW3_RS0113260	WP_010222978.1	triose-phosphate isomerase
Scaffold 8 199986-199987	UW3_RS0113375	WP_010222999.1	sigma-54-dependent Fis family transcriptional regulator
Scaffold 8 201794-201795	UW3_RS0113380	WP_036995606.1	PAS domain S-box protein
Scaffold 8 219597-219598	UW3_RS0113470	WP_010223017.1	ketol-acid reductoisomerase
Scaffold 9 47174-47175	UW3_RS0113685	WP_010223062.1	ATP-dependent helicase
Scaffold 9 114849-114850	UW3_RS0114040	WP_010223132.1	ribonuclease D
Scaffold 9 132657-132658	UW3_RS0114110	WP_010223149.1	sensor histidine kinase
Scaffold 10 27163-27164	UW3_RS0114525	WP_026001332.1	endolytic transglycosylase MltG
Scaffold 10 140011-140012	UW3_RS0115035	WP_010223327.1	quinone-dependent dihydroorotate dehydrogenase
Scaffold 10 172925-172926	UW3_RS0115160	WP_010223355.1	ABC transporter ATP-binding protein
Scaffold 10 177607-177608	UW3_RS0115175	WP_010223358.1	hypothetical protein
Scaffold 10 182211-182212	UW3_RS0115205	WP_010223363.1	mannose-1-phosphate guanylyltransferase/mannose-6-phosphate isomerase
Scaffold 11 16255-16256	UW3_RS0115340	WP_010223391.1	superoxide dismutase
Scaffold 11 20916-20917	UW3_RS0115370	WP_010223396.1	ribosome-associated protein
Scaffold 11 130603-130604	UW3_RS0115915	WP_010223506.1	alanine transaminase
Scaffold 12 10874-10875	UW3_RS0116200	WP_010223562.1	thiolase domain-containing protein
Scaffold 13 77321-77322	UW3_RS0117230	WP_010223914.1	UTP-glucose-1-phosphate uridylyltransferase
Scaffold 14 36444-36445	UW3_RS0117890	WP_010224127.1	aminotransferase
Scaffold 14 78741-78742	UW3_RS0118080	WP_010224179.1	hypothetical protein
Scaffold 14 105154-105155	UW3_RS0118215	WP_010224220.1	NAD-dependent epimerase
Scaffold 14 113810-113811	UW3_RS0118255	WP_036995848.1	hypothetical protein
Scaffold 15 14510-14511	UW3_RS0118455	WP_010224295.1	monovalent cation/H+ antiporter subunit A
Scaffold 15 32640-32641	UW3_RS0118535	WP_010224326.1	enoyl-CoA hydratase
Scaffold 15 128079-128080	UW3_RS0118995	WP_010224685.1	citrate (Si)-synthase
Scaffold 16 45141-45142	UW3_RS0119320	WP_010224806.1	RNA polymerase sigma factor RpoS
Scaffold 17 24341-24342	UW3_RS0119825	WP_010224957.1	protein-glutamate O-methyltransferase CheR
Scaffold 17 26972-26973	UW3_RS0119840	WP_010224961.1	flagellar hook assembly protein FlgD
Scaffold 18 64146-64147	UW3_RS0120620	WP_010225229.1	cytochrome-c oxidase, cbb3-type subunit I

Insertion site	Identity	Protein ID	Protein
Scaffold 18 67336-67337	UW3_RS0120630	WP_010225232.1	acyl-CoA synthetase
Scaffold 18 69733-69734	UW3_RS0120640	WP_010225234.1	ribulose-phosphate 3-epimerase
Scaffold 18 84878-84879	UW3_RS0120700	WP_010225250.1	phenylacetate-CoA oxygenase/reductase subunit PaaK
Scaffold 19 58445-58446	UW3_RS0121165	WP_010225412.1	DNA-binding response regulator
Scaffold 20 98472-98473	UW3_RS0121975	WP_010225680.1	LysR family transcriptional regulator
Scaffold 22 2011-2012	UW3_RS0122005	WP_010225700.1	Obg family GTPase CgtA
Scaffold 25_1 16733-16734	UW3_RS0100440	WP_010220156.1	2-isopropylmalate synthase
Scaffold 25_1 60431-60432	UW3_RS0100620	WP_010220196.1	glycine dehydrogenase (aminomethyl-transferring)
Scaffold 25_1 71118-71119	UW3_RS0100670	WP_010220208.1	valine-tRNA ligase
Scaffold 33_1 32575-32576	UW3_RS0101485	WP_026001148.1	hybrid sensor histidine kinase/response regulator
Scaffold 35 27383-27384	UW3_RS0124905	WP_010226656.1	arginine N-succinyltransferase
Scaffold 38 24892-24893	UW3_RS0125385	WP_010226811.1	anti-sigma factor
Scaffold 56_1 709-710	UW3_RS0102220	WP_010220558.1	membrane-bound lytic murein transglycosylase MltF

Supplementary Table S2. Screened genes classified by pathways

Insertion site	Identity	Pathway	Protein
Scaffold 2 204840-204841	WP_036995327.1	1	hypothetical protein
Scaffold 2 291005-291006	WP_010221378.1	1	pyruvate dehydrogenase (acetyl-transferring), homodimeric type
Scaffold 2 332202-332203	WP_026001208.1	1	MOSC domain-containing protein
Scaffold 3 35451-35452	WP_010221513.1	1	methylenetetrahydrofolate reductase [NAD(P)H]
Scaffold 3 128460-128461	WP_010221593.1	1	tRNA (adenosine(37)-N6)-dimethylallyl transferase MiaA
Scaffold 3 270866-270867	WP_010221729.1	1	methyltransferase domain-containing protein
Scaffold 3 298275-298276	WP_010221756.1	1	methylmalonate-semialdehyde dehydrogenase (CoA acylating)
Scaffold 4 32850-32851	WP_010221794.1	1	CoA transferase subunit A
Scaffold 7 69348-69349	WP_010222657.1	1	oligopeptidase A
Scaffold 8 173223-173224	WP_010222978.1	1	triosephosphate isomerase
Scaffold 8 219597-219598	WP_010223017.1	1	ketol-acid reductoisomerase
Scaffold 10 140011-140012	WP_010223327.1	1	quinone-dependent dihydroorotate dehydrogenase

Insertion site	Identity	Pathway	Protein
Scaffold 10 182211-182212	WP_010223363.1	1	mannose-1-phosphate guanylyltransferase/mannose-6-phosphate isomerase
Scaffold 11 16255-16256	WP_010223391.1	1	superoxide dismutase
Scaffold 11 130603-130604	WP_010223506.1	1	alanine transaminase
Scaffold 12 10874-10875	WP_010223562.1	1	thiolase domain-containing protein
Scaffold 13 77321-77322	WP_010223914.1	1	UTP-glucose-1-phosphate uridylyltransferase
Scaffold 14 36444-36445	WP_010224127.1	1	aminotransferase
Scaffold 14 105154-105155	WP_010224220.1	1	NAD-dependent epimerase
Scaffold 15 32640-32641	WP_010224326.1	1	enoyl-CoA hydratase
Scaffold 15 128079-128080	WP_010224685.1	1	citrate (Si)-synthase
Scaffold 18 64146-64147	WP_010225229.1	1	cytochrome-c oxidase, cbb3-type subunit I
Scaffold 18 67336-67337	WP_010225232.1	1	acyl-CoA synthetase
Scaffold 18 69733-69734	WP_010225234.1	1	ribulose-phosphate 3-epimerase
Scaffold 18 84878-84879	WP_010225250.1	1	phenylacetate-CoA oxygenase/reductase subunit PaaK
Scaffold 22 2011-2012	WP_010225700.1	1	Obg family GTPase CgtA
Scaffold 25_1 16733-16734	WP_010220156.1	1	2-isopropylmalate synthase
Scaffold 25_1 60431-60432	WP_010220196.1	1	glycine dehydrogenase(aminomethyl-transferring)
Scaffold 35 27383-27384	WP_010226656.1	1	arginine N-succinyltransferase
Scaffold 1 38526-38527	WP_010220635.1	2	amino acid metabolite efflux pump
Scaffold 1 53875-53876	WP_010220648.1	2	PAS domain S-box protein
Scaffold 1 62596-62597	WP_010220659.1	2	TonB-dependent siderophore receptor
Scaffold 2 159889-159890	WP_010221262.1	2	outer membrane porin, OprD family
Scaffold 2 185441-185442	WP_026001200.1	2	type VI secretion system lipoprotein TssJ
Scaffold 3 188651-188652	WP_010221650.1	2	urea ABC transporter ATP-binding protein UrtD
Scaffold 4 115279-115280	WP_010221877.1	2	two-component system response regulator UvrY
Scaffold 4 204936-204937	WP_010221972.1	2	protein-glutamate O-methyltransferase CheR
Scaffold 5 9638-9639	WP_010222066.1	2	ABC transporter substrate-binding protein
Scaffold 5 155604-155605	WP_010222233.1	2	cell division protein
Scaffold 8 199986-199987	WP_010222999.1	2	sigma-54-dependent Fis family transcriptional regulator
Scaffold 8 201794-201795	WP_036995606.1	2	PAS domain S-box protein

Insertion site	Identity	Pathway	Protein
Scaffold 9 132657-132658	WP_010223149.1	2	sensor histidine kinase
Scaffold 10 172925-172926	WP_010223355.1	2	ABC transporter ATP-binding protein
Scaffold 12 10874-10875	WP_010223562.1	2	thiolase domain-containing protein
Scaffold 14 113810-113811	WP_036995848.1	2	hypothetical protein
Scaffold 15 14510-14511	WP_010224295.1	2	monovalent cation/H+ antiporter subunit A
Scaffold 17 24341-24342	WP_010224957.1	2	protein-glutamate O-methyltransferase CheR
Scaffold 18 64146-64147	WP_010225229.1	2	cytochrome-c oxidase, cbb3-type subunit I
Scaffold 33_1 32575-32576	WP_026001148.1	2	hybrid sensor histidine kinase/response regulator
Scaffold 56_1 709-710	WP_010220558.1	2	membrane-bound lytic murein transglycosylase MltF
Scaffold 1 348413-348414	WP_010220971.1	3	RNA polymerase sigma factor RpoE
Scaffold 2 346446-346447	WP_026001209.1	3	transcriptional regulator
Scaffold 3 128460-128461	WP_010221593.1	3	tRNA (adenosine(37)-N6)-dimethylallyl transferase MiaA
Scaffold 3 221405-221406	WP_010221683.1	3	tRNA dihydrouridine synthase DusB
Scaffold 3 270866-270867	WP_010221729.1	3	methyltransferase domain-containing protein
Scaffold 4 115279-115280	WP_010221877.1	3	two-component system response regulator UvrY
Scaffold 5 186598-186599	WP_010222267.1	3	thioredoxin TrxC
Scaffold 6 236951-236952	WP_010222573.1	3	hypothetical protein
Scaffold 9 47174-47175	WP_010223062.1	3	ATP-dependent helicase
Scaffold 9 114849-114850	WP_010223132.1	3	ribonuclease D
Scaffold 11 20916-20917	WP_010223396.1	3	ribosome-associated protein
Scaffold 16 45141-45142	WP_010224806.1	3	RNA polymerase sigma factor RpoS
Scaffold 19 58445-58446	WP_010225412.1	3	DNA-binding response regulator
Scaffold 20 98472-98473	WP_010225680.1	3	LysR family transcriptional regulator
Scaffold 22 2011-2012	WP_010225700.1	3	Obg family GTPase CgtA
Scaffold 25_1 71118-71119	WP_010220208.1	3	valine-tRNA ligase
Scaffold 38 24892-24893	WP_010226811.1	3	anti-sigma factor
Scaffold 1 53875-53876	WP_010220648.1	4	PAS domain S-box protein
Scaffold 4 115279-115280	WP_010221877.1	4	two-component system response regulator UvrY
Scaffold 4 204936-204937	WP_010221972.1	4	protein-glutamate O-methyltransferase CheR

Insertion site	Identity	Pathway	Protein
Scaffold 10 27163-27164	WP_026001332.1	4	endolytic transglycosylase MltG
Scaffold 10 182211-182212	WP_010223363.1	4	mannose-1-phosphate guanylyltransferase/mannose-6-phosphate isomerase
Scaffold 17 26972-26973	WP_010224961.1	4	flagellar hook assembly protein FlgD
Scaffold 33_1 32575-32576	WP_026001148.1	4	hybrid sensor histidine kinase/response regulator
Scaffold 3 118890-118891	WP_010221584.1	5	TIR domain-containing protein
Scaffold 3 35451-35452	WP_010221513.1	6	methylenetetrahydrofolate reductase [NAD(P)H]
Scaffold 6 241825-241826	WP_010222576.1	7	phosphoesterasePHP domain-containing protein
Scaffold 7 150642-150643	WP_010222750.1	7	hypothetical protein
Scaffold 10 177607-177608	WP_010223358.1	7	hypothetical protein
Scaffold 14 78741-78742	WP_010224179.1	7	hypothetical protein

Note: 1, Metabolism. 2, Environmental Information Processing. 3, Genetic Information Processing. 4, Cellular Processes. 5, Organismal Systems. 6, Human Diseases. 7, Uncharacterized Protein.

Supplementary Table S3. Identification of *UW3_RS0113375* and *UW3_RS0113380*

Gene site	No. of amino acid residues	Protein	Organism compared	% similarity/ % identity	% coverage	Significance (E value)
UW3_RS0113375	479	CbrB	*P. aeruginosa* PA14/PAO1	90/85	99	0.0
UW3_RS0113380	982	CbrA	*P. aeruginosa* PA14/PAO1	92/83	99	0.0

Supplementary Table S4. The potential virulence-related genes screened were regulated by the Cbr/Crc two-component system

1. Transcription levels of genes in the *ΔcbrA* strain

Gene	Upregulated				Gene	Downregulated			
	Fold Change		FDR	P-value		Fold Change		FDR	P-value
UW3_RS0105350	1.76	Up	1.16E-191	3.59E-193	UW3_RS0107755	2.73	Down	2.86E-88	1.66E-89
UW3_RS0105440	1.57	Up	5.41E-11	1.34E-11	UW3_RS0108730	1.61	Down	5.04E-07	1.61E-07
UW3_RS0111395	1.53	Up	1.62E-02	8.64E-03	UW3_RS0112125	1.64	Down	2.89E-15	6.03E-16
UW3_RS0113260	2.32	Up	3.29E-84	2.02E-85	UW3_RS0115340	6.4	Down	3.47E-248	7.73E-250
UW3_RS0113685	1.65	Up	1.97E-16	3.86E-17	UW3_RS0116200	3.26	Down	1.04E-55	9.01E-57
UW3_RS0114525	1.52	Up	6.29E-05	2.41E-05	UW3_RS0120700	1.55	Down	1.52E-07	4.68E-08
UW3_RS0115035	1.55	Up	5.26E-14	1.16E-14	UW3_RS0107620	1.48	Down	0.000108	0.0000423
UW3_RS0115175	2.28	Up	1.40E-02	7.35E-03	UW3_RS0110010	1.33	Down	0.0309	0.0173
UW3_RS0115915	2.05	Up	9.98E-35	1.20E-35	UW3_RS0111420	1.36	Down	9.70E-10	2.59E-10
UW3_RS0117890	1.68	Up	1.90E-16	3.71E-17	UW3_RS0125385	1.41	Down	0.0301	0.0168
UW3_RS0118080	1.98	Up	4.43E-05	1.67E-05					
UW3_RS0118215	1.69	Up	2.52E-232	6.30E-234					
UW3_RS0118255	1.57	Up	6.96E-63	5.38E-64					
UW3_RS0118995	3.56	Up	0	0					
UW3_RS0120630	1.83	Up	1.12E-63	8.57E-65					
UW3_RS0120640	3.23	Up	1.35E-77	8.95E-79					
UW3_RS0121165	1.76	Up	1.59E-02	8.48E-03					

Gene	Upregulated				Gene	Downregulated		
	Fold Change		FDR	P-value		Fold Change	FDR	P-value
UW3_RS0122005	2.07	Up	5.87E-43	6.02E-44				
UW3_RS0100620	9.9	Up	0	0				
UW3_RS0124905	3.02	Up	1.37E-85	8.32E-87				
UW3_RS0106995	1.41	Up	1.34E-14	2.85E-15				
UW3_RS0100670	1.44	Up	1.60E-42	1.67E-43				

2. Transcription levels of genes in the *ΔcbrB* strain

Gene	Upregulated			
	Fold Change		FDR	P-value
UW3_RS0106110	4.2	Up	4.52E-33	4.06E-34
UW3_RS0107920	1.57	Up	1.45E-07	3.51E-08
UW3_RS0111745	1.51	Up	6.70E-40	5.19E-41
UW3_RS0119840	1.56	Up	1.78E-11	3.34E-12
UW3_RS0100440	1.69	Up	2.83E-75	1.38E-76
UW3_RS0102220	1.8	Up	1.96E-14	3.18E-15

3. Transcription levels of genes in the *ΔcrcZΔcrcY* strain

Gene	Upregulated				Gene	Downregulated			
	Fold Change		FDR	P-value		Fold Change		FDR	P-value
UW3_RS0118535	1.81	Up	9.11E-72	5.09E-73	UW3_RS0105940	1.61	Down	1.14E-95	5.18E-97
					UW3_RS0113470	4.14	Down	0	0
					UW3_RS0117230	1.59	Down	1.99E-24	2.09E-25
					UW3_RS0119825	1.17	Down	0.00622	0.00234

4. Transcription levels of genes in the *crc*-overexpression strain

Gene	Upregulated				Gene	Downregulated			
	Fold Change		FDR	P-value		Fold Change		FDR	P-value
UW3_RS0102520	4.03	Up	2.78E-59	3.11E-60	UW3_RS0119320	3.62	Down	0	0
UW3_RS0102580	2.54	Up	2.03E-33	3.73E-34	UW3_RS0120620	1.83	Down	2.98E-73	2.76E-74
UW3_RS0103900	2.1	Up	2.38E-114	1.57E-115	UW3_RS0108300	1.21	Down	0.00242	0.00162
UW3_RS0105515	2.66	Up	5.05E-14	1.71E-14	UW3_RS0115370	1.39	Down	0.000398	0.000246
UW3_RS0106175	1.75	Up	2.44E-08	1.11E-08					
UW3_RS0106600	1.66	Up	1.52E-18	4.27E-19					
UW3_RS0106950	3.47	Up	1.69E-20	4.48E-21					
UW3_RS0107275	2.6	Up	1.07E-05	5.78E-06					
UW3_RS0107430	1.95	Up	2.94E-62	3.15E-63					
UW3_RS0109160	3.16	Up	1.78E-18	5.06E-19					
UW3_RS0114040	1.54	Up	4.16E-16	1.29E-16					
UW3_RS0114110	1.85	Up	1.25E-30	2.45E-31					
UW3_RS0115205	3.59	Up	8.77E-64	9.30E-65					

Gene	Upregulated				Gene	Downregulated		
	Fold Change		FDR	P-value		Fold Change	FDR	P-value
UW3_RS0102620	1.45	Up	1.69E-11	6.47E-12				
UW3_RS0109860	1.32	Up	9.02E-25	2.09E-25				
UW3_RS0115160	1.34	Up	2.44E-06	1.26E-06				
UW3_RS0118455	1.45	Up	3.24E-11	1.25E-11				
UW3_RS0121975	1.31	Up	0.0103	0.00741				
UW3_RS0101485	1.34	Up	1.28E-11	4.87E-12				

Supplementary Table S5. Bacterial strains and plasmids used in this study

Strains and Plasmids	Description	Reference
Strain		
E. coli		
S17-1 λ*pir*	*recA*, *thi*, *pro*, $hsdR^{-}M^{+}$, Sm^{R}, <RP4:2-Tc:Mu:Ku:Tn7> Tp^{R}	Simon et al., 1983
P. donghuensis		
HYS	Wild-type, lethal to *C. elegans*, Cm^{r}	Preserved in our laboratory
HYS1	*ΔcbrA*	This study
HYS2	*ΔcbrB*	This study
HYS3	*ΔcrcZ*	This study
HYS4	*ΔcrcY*	This study
HYS5	*Δcrc*	This study
HYS6	*ΔcrcZΔcrcY*	This study
HYS7	*ΔcbrAΔcrc*	This study
HYS8	*ΔcbrBΔcrc*	This study
HYS9	*ΔcbrA*/pBBR1MCS-2	This study
HYS10	*ΔcbrA*/pBBR2-*cbrA*	This study
HYS11	*ΔcbrB*/pBBR1MCS-2	This study
HYS12	*ΔcbrB*/pBBR2-*cbrAB*	This study
HYS13	*ΔcbrAΔcrc*/pBBR1MCS-2	This study
HYS14	*ΔcbrAΔcrc*/pBBR2-*crc*	This study
HYS15	*ΔcbrBΔcrc*/pBBR1MCS-2	This study
HYS16	*ΔcbrBΔcrc*/pBBR2-*crc*	This study
HYS17	HYS/pBBR1MCS-2	This study
HYS18	HYS/pBBR2-*crc*	This study
Plasmids		
pBT20	Mariner transposon mutagenesis vector, Ap^{r}, Gm^{r}	Kulasekara et al., 2005
pEX18Gm	Gene replacement vector, Gm^{r}, $oriT^{+}$, $sacB^{+}$	Hoang et al., 1998
pEX18Gm-*cbrA*-UD	Gene replacement vector for *cbrA*	This study
pEX18Gm-*cbrB*-UD	Gene replacement vector for *cbrB*	This study
pEX18Gm-*crcZ*-UD	Gene replacement vector for *crcZ*	This study
pEX18Gm-*crcY*-UD	Gene replacement vector for *crcY*	This study
pEX18Gm-*crc*-UD	Gene replacement vector for *crc*	This study
pBBR1MCS-2	Mobilizable broad-host-range cloning vector, Km^{r}	Kovach et al., 1995
pBBR2-*cbrAB*	Cloning vector for *cbrAB*	This study
pBBR2-*cbrA*	Cloning vector for *cbrA*	This study
pBBR2-*crc*	Cloning vector for *crc*	This study

Abbreviations of antibiotics: Cm, chloramphenicol. Gm, gentamicin. Km, kanamycin.

References

Simon et al., 1983: *Bio-Technology* 1 (9): 784-791.

Kulasekara et al., 2005: *Mol Microbiol* 55 (2): 368-380.

Hoang et al., 1998: *Gene* 212 (1): 77-86.

Kovach et al., 1995 *Gene* 166 (1): 175-176.

Supplementary Table S6. Primers used in this study

Name	Sequence 5′-3′	Purpose
cbrA-up-1	CGGGGTACCAAAGCTCATCGGCATC	Construction of *ΔcbrA*
cbrA-up-2	CGGAATTCACTGGGCAAGTCCTACC	Construction of *ΔcbrA*
cbrA-down-1	GCTCTAGAGCGCGACCTTGCGTATC	Construction of *ΔcbrA*
cbrA-down-2	CGGGGTACCGTGAACTGAGAC	Construction of *ΔcbrA*
cbrA-M-1	GAGATCATCGGTGCCTTGG	Verification of *ΔcbrA*
cbrA-M-2	GCGCCAGCTGTATCTGCA	Verification of *ΔcbrA*
cbrB-up-1	CGGGGTACCATGCGGCATTGATTC	Construction of *ΔcbrB*
cbrB-up-2	CGGAATTCGGCAGCCACCAGCAC	Construction of *ΔcbrB*
cbrB-down-1	GCTCTAGAAGAAGTAGTTGCGTGCT	Construction of *ΔcbrB*
cbrB-down-2	CGGGGTACCTGAGCCGTTTC	Construction of *ΔcbrB*
cbrB-M-1	TAGCATTTACTCGGAGGATGG	Verification of *ΔcbrB*
cbrB-M-2	CTTGCCTGGCACAGAACC	Verification of *ΔcbrB*
crcZ-up-1	CGGGGTACCACCAAACATATAGCAG	Construction of *ΔcrcZ*
crcZ-up-2	CGGAATTCAGCTGGAAAACGCC	Construction of *ΔcrcZ*
crcZ-down-1	GCTCTAGACCGCCCACTCTCGTTAC	Construction of *ΔcrcZ*
crcZ-down-2	CAAGGTACCCCGACGCGACCGCCTA	Construction of *ΔcrcZ*
crcZ-M-1	CTCGCTGACCGGGTCGTAA	Verification of *ΔcrcZ*
crcZ-M-2	AAATCGCCAACGCCTTCC	Verification of *ΔcrcZ*
crcY-up-1	CAGAATTCGCCTGTTCGAAGTGGT	Construction of *ΔcrcZ*
crcY-up-2	CGCGGTACCCTGAGATATAGCAG	Construction of *ΔcrcY*
crcY-down-1	CAAGGTACCCCTTCTCAAAGCGATTG	Construction of *ΔcrcY*
crcY-down-2	GCTCTAGATCGAATAATCCCTCCTGC	Construction of *ΔcrcY*
crcY-M-1	GTGTCCTGCGTGCTGGGTTG	Verification of *ΔcrcY*
crcY-M-2	GGTCTGGTCAATTTCAATAGGC	Verification of *ΔcrcY*
crc-up-1	CGGAATTCAGTACGCCAGCAGCCTT	Construction of *Δcrc*
crc-up-2	CAAGGTACCCCGCATAAATGGC	Construction of *Δcrc*
crc-down-1	CGGGGTACCACGCTGACCATCTGAT	Construction of *Δcrc*
crc-down-2	GCTCTAGAGGCGCAAAAGGGCATGA	Construction of *Δcrc*
crc-M-1	ACGATGCTCACCACCGGAATG	Verification of *Δcrc*
crc-M-2	AAGACTCGGCCGCCGAGACC	Verification of *Δcrc*
cbrAB-up/EcoR	GAGAATTCCAGAGCCAACGTGATGC	Amplification of *cbrAB* and *cbrA*
cbrAB-down/XbaF	GCTCTAGAAACGGCTCAGCCCTCG	Amplification of *cbrAB*
cbrA-down/XbaF	GCTCTAGAACGGTCTCAGTTCACAGC	Amplification of *cbrA*
crc-up/EcoF	GAGAATTCGGCTGCATGAAGAGTC	Amplification of *crc*
crc-down/XbaR	GCTCTAGACGGCTTTTTGTGGCTG	Amplification of *crc*
